# Explaining cancer type specific mutations with transcriptomic and epigenomic features in normal tissues

**DOI:** 10.1038/s41598-018-29861-1

**Published:** 2018-07-30

**Authors:** Khong-Loon Tiong, Chen-Hsiang Yeang

**Affiliations:** Institute of Statistical Science, Academia Sinica, Taipei, Taiwan

## Abstract

Most cancer driver genes are involved in generic cellular processes such as DNA repair, cell proliferation and cell adhesion, yet their mutations are often confined to specific cancer types. To resolve this paradox, we explained mutation frequencies of selected genes across tumor types with four features in the corresponding normal tissues from cancer-free subjects: mRNA expression and chromatin accessibility of mutated genes, mRNA expressions of their neighbors in curated pathways and the protein-protein interaction network. Encouragingly, these transcriptomic/epigenomic features in normal tissues were closely associated with mutational/functional characteristics in tumors. First, chromatin accessibility was a necessary but not sufficient condition for frequent mutations. Second, variations of mutation frequencies in selected genes across tissue types were significantly associated with all four features. Third, the genes possessing significant associations between mutation frequency variations and pathway gene expression were enriched with documented cancer genes. We further proposed a novel bivariate gene set enrichment analysis and confirmed that the pathway gene expression was the dominant factor in cancer gene enrichment. These findings shed lights on the functional roles of genes in normal tissues in shaping the mutational landscape during tumor genome evolution.

## Introduction

Cancer cells harbor a large number of mutations on diverse genes with a wide range of occurrence frequencies^1^. Mutations on “driver” genes alter important cellular processes – such as the “hallmarks” like cell proliferation, apoptosis and angiogenesis – and thus drive oncological phenotypes such as tumorigenesis, progression and metastasis^2,3^. Mutations on the vast majority of “passenger” genes, in contrast, are likely the byproducts of unstable cancer genomes and bear little or no phenotypical consequences^4,5^. Much of the past and recent progress in cancer research has been on charting the mutational landscapes of cancers and deciphering their functional implications. Pre-genomic studies have already mapped recurrent mutations on various driver genes and confirmed their oncological roles^6–9^. High-throughput sequencing technologies further advanced the knowledge about the prevalence and functions of the well-known driver mutations^10–13^, and mapped rare mutations in large populations^14–16^. Today, our knowledge about the mutational landscapes of human cancers is likely near complete and accessible to the general public, thanks to the efforts of converting decades of studies into structured databases^17,18^ and several international consortia generating large-scale cancer genomic data^19–25^. There is also tremendous progress in identifying driver genes and deciphering their functions, thanks to various bioinformatics tools^26–28^ and interventional technologies such as shRNAs and CRISPR^29–32^.

Despite the progress in these directions, major puzzles persist in cancer genomics. One of the unresolved paradoxes is the reconciliation between tumor specific mutations and generic functions of the mutated genes. Mutations of most well-known driver genes are confined primarily to certain cancer types, such as APC and beta catenin in colorectal cancers^20,33,34^; BRCA1 and BRCA2 in breast and ovarian cancers^19,22,35^; BRAF in melanoma, thyroid and colorectal cancers^36–38^; FLT3 and CEBPA in AML^39,40^; EGFR in non-small cell lung cancers and brain tumors^41,42^. Perhaps the only two exceptions are TP53 and KRAS, which are frequently mutated in most cancer types^43,44^. Intriguingly, many frequently mutated genes are primarily involved in fundamental biological processes such as DNA repair^45^, cell cycle regulation^46^, cell adhesion^47^, processes which are important in cancer^2,3^ as well as most normal tissues. In the aforementioned examples, APC and beta catenin are members of the Wnt pathway^34^; EGFR, BRAF and FLT3 are members of the Ras pathway^48–50^; BRCA1 and BRCA2 are involved in DNA repair^45^. Those processes are essential for the development and maintenance of almost all tissue types. It is quite puzzling why their mutations are observed in specific cancer types.

Recurrent mutations are consequences of tumor clonal evolution. Mutations that enhance fitness of cells are fixed in the expanding subclones and thus appear frequently in the patients^51–53^. Tissue-specific mutations, therefore, likely reflect tissue-specific selective advantages of the mutant cells. It is very difficult, if possible, to directly observe clonal evolution and measure the selective advantages of specific mutations. As a substitute for direct observations of clonal evolution, we suspect that key evidence of selective advantages of mutations exists in the features of normal tissues. For instance, if a gene is not accessible for transcription in a specific tissue, then it may not be transcribed and play any significant role in that tissue. Mutations on the gene thus confer no selective advantage and do not appear frequently in the population.

This line of reasoning motivated us to investigate the roles of features in normal tissues in explaining cancer type-specific mutations of genes undergoing selection in cancer genome evolution. In particular, we considered four transcriptomic and epigenomic features in normal tissues: chromatin accessibility and expressions of the targeted genes, expressions of documented pathways and protein binding partners of the targeted genes. Those features were extracted from the data of cancer-free subjects, thus can better reflect pre-cancerous conditions than the data from normal tissues adjacent to tumors. For each candidate feature, we associated variations of mutation frequencies with variations of feature values over multiple tissue types. In addition, we checked whether associations with single or double features revealed the cancer-related functions of genes. Favorably, our analysis outcomes suggest features in normal tissues contain information about tissue-specific mutations and oncological functions of genes, despite the fact that the data of cancers and normal tissues were collected from completely independent sources. The encouraging results serve as an early step toward understanding molecular and clinical characteristics of cancers from an evolutionary perspective.

Various previous studies aimed for explaining patterns of gene mutations from evolutionary and environmental perspectives. Schaefer and Serrano categorized mutated genes into tissue-general and tissue-specific groups and examined their distinctions in gene expressions, functional annotations, and environmental interactions^54^. Lim *et al*. concluded that active histone marks were enriched around frameshift indels as compared to missense single nucleotide variation, while repressive histone marks showed the opposite trend^55^. Yeang *et al*. showed co-occurring mutations happened at genes in distinct pathways and mutually exclusive mutations happened at genes in the same pathways, hence indirectly validated the clonal evolution hypothesis^56^. Lawrence *et al*. showed the spectrum of sequence mutations was closely linked with carcinogenic factors^57^. Our work distinguishes from those prior studies as it systematically explains cancer type-specific mutations with features in normal tissues. Several recent studies tackled similar problems of explaining cancer type-specific mutations with genetic and epigenetic features in normal tissues. Polak *et al*. showed that variance in mutation rates along cancer genomes was largely explained by cell-of-origin epigenomic states of independent normal samples^58^. Ha *et al*. discovered that the somatic mutational landscape in cancer was associated with chromatin features in the corresponding pre-cancerous tissues, which were altered by environmental exposure^59^. Yamashita *et al*. demonstrated that genetic and epigenetic alterations in normal tissues had different effects on cancer risk between gastric and esophageal cancers^60^. Our work shares the common goal with these studies yet adopts different approaches. We included both epigenomic and transcriptomic features, while prior analysis examined exclusively on either epigenomic states or gene expressions alone. Furthermore, we performed association analysis both within each cancer type and across multiple cancer types, while most prior studies targeted within-tissue analysis.

## Results

### Chromatin accessibility in normal tissues is a necessary but not sufficient condition for mutations in tumors

**Figure 1:**
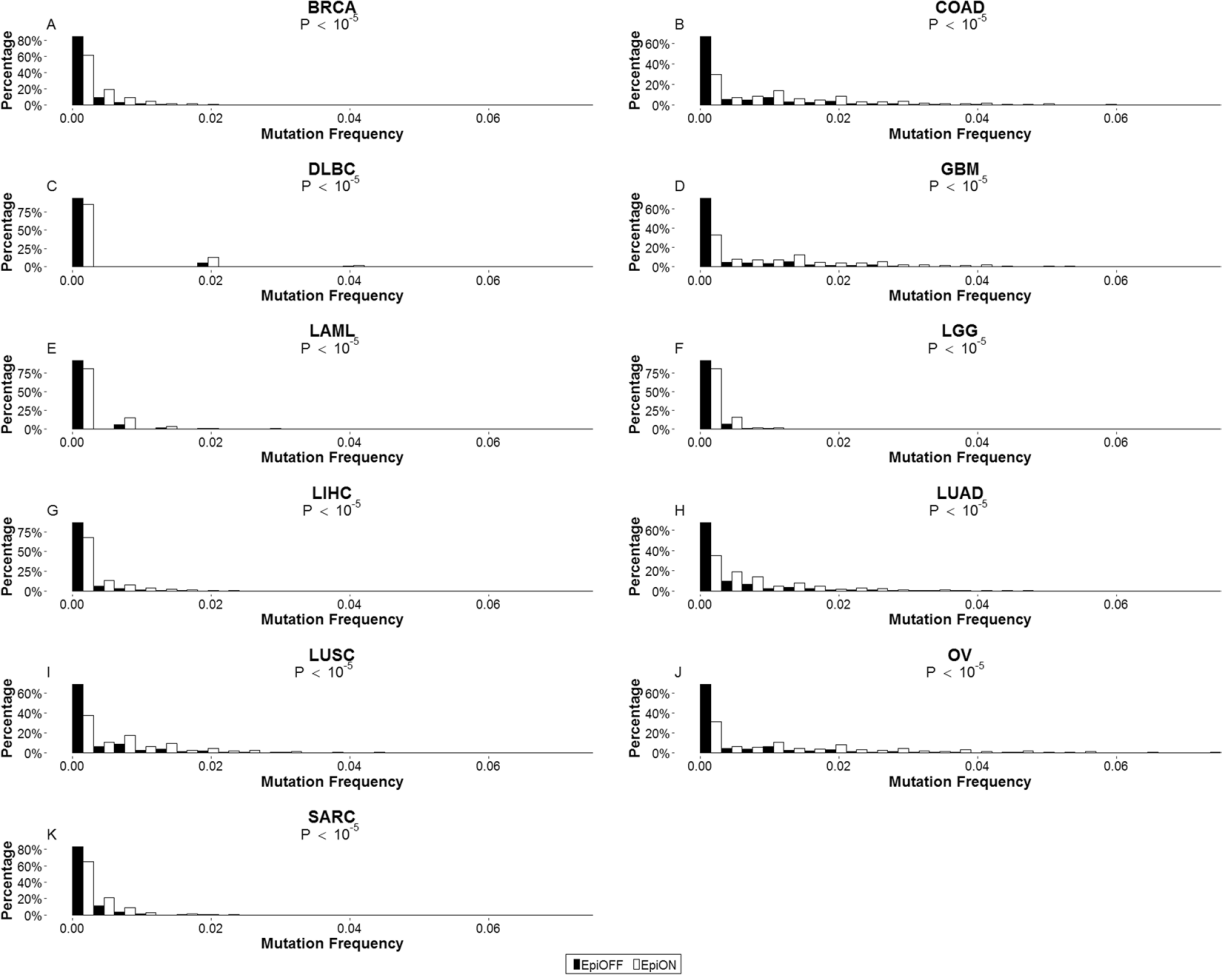
Tissue-specific distributions of mutation frequency of EpiON and EpiOFF genes. EpiON genes were genes classified as chromatin-accessible for transcription, while EpiOFF genes were classified as chromatin-inaccessible for transcription. Each panel shows the distributions of mutation frequencies of EpiON (white bars) and EpiOFF (black bars) genes in the designated cancer type (p-values < 10^−5^ for all cancer types according to permutation tests).

Chromatin accessibility is a fundamental requisite for transcription. A gene which is not accessible at the chromatin level is in principle not amenable for further downstream regulation and thus confers no functional roles in the corresponding normal tissue. For each tissue type, we divided genes into “EpiON” and “EpiOFF” groups according to their chromatin accessibility states. The EpiON genes possess chromatin states accessible for active transcription, and the EpiOFF genes have inaccessible chromatin states. We compared the distributions of mutation frequencies between the two groups in 11 cancer types (Fig. 1). Among all the cancer types examined, the EpiON genes consistently possessed significantly higher mutation frequencies than the EpiOFF genes (p-values < 10^−5^ for all cancer types according to permutation tests). The distribution of EpiOFF genes was sharply peaked around 0, indicating that they were rarely mutated in the corresponding tumor types. In contrast, the distribution of EpiON genes had a much wider tail, indicating diverse mutation frequencies of the members.

The distributions of mRNA expressions in normal tissues between EpiON and EpiOFF genes (Fig. S1; permutation p-values < 10^−5^ for all cancer types) exhibit a similar but weaker pattern as Fig. 1: the EpiOFF genes were hardly expressed, whereas the EpiON genes possessed a wider range of expression levels. However, when dividing genes in terms of their expressions in normal tissues, the mutation frequency distributions of the highly expressed and lowly expressed groups did not possess pronounced or consistent differences. This property holds for a wide range of threshold values determining highly or lowly expressed genes (Figs S2–S4). These results suggest that chromatin accessibility in normal tissues is a necessary but not sufficient condition for mutations in cancer, whereas expression levels within each tissue type have a much weaker correlation with mutation frequencies.

### Variations of mutation frequencies across cancer types are associated with epigenomic/transcriptomic features in normal tissues

The aforementioned tissue-specific studies motivated a more thorough investigation about the relations between mutational profiles across cancer types and various normal tissue features. For each gene, we constructed a vector of mutational profile over available cancer types, where each component indicates its mutation frequency $(\frac{\#\,mutated\,samples}{\#\,samples})$ in a specific cancer type. Our aim was to identify features in normal tissues that were associated with the mutational profiles of selected genes.

Chromatin accessibility and mRNA expressions are obvious candidate features to fit their mutational profiles. The indistinguishable distributions of mutation frequencies between highly expressed and lowly expressed genes within each cancer type (Figs S2–S4) do not preclude the possible association between mutational profiles and gene expressions across tissue types. Beyond expression and chromatin states, the function of a gene can also be characterized by activities of other genes participating in the same biological processes. We quantified the contextual information of a gene with two additional features in normal tissues: expressions of the selected gene’s protein-protein interaction partners (inferred protein-binding partner activities) and immediate upstream/downstream members in the curated pathways (inferred pathway activities). These contextual genes are first-degree neighbors of the selected gene in the networks spanned by protein-protein interactions and curated pathways respectively. To assess the effectiveness of associations of those features, we introduced the fifth control feature by randomly selecting the third to seventh-degree neighbors of the selected gene in the protein-protein interaction network and averaging their expression levels. The third to seventh degree neighbors are remotely connected to the selected gene and thus are anticipated to have weak influence comparable to the background.

Mutations of functionally relevant genes are likely to occur with high frequencies since they confer selective advantages during tumor clonal evolution. We considered the candidate genes undergoing recurrent mutations by combining the top-50 most mutated genes from all cancer types to a list of 432 genes. This list is substantially smaller than the genes in the tissue-specific analysis (Fig. 1), which basically cover all genes possessing both epigenomic and mutation data. This is because the cross-tissue analysis required genes with varying mutational profiles (i.e., frequently mutated in some cancer types and rarely or not mutated in others), and very few genes were frequently mutated across all cancer types investigated. In contrast, the tissue-specific analysis considered each tissue type independently, thus included a large number of genes with low or zero mutation frequencies across all cancer types.

A differentially mutated gene typically has high mutation frequencies in one or two cancer types and low or zero mutation frequencies in the remaining cancer types. Furthermore, the mutation frequency gap between the highly and lowly/non-mutated groups is often prominent. In contrast, transcriptomic features of a gene typically manifest a continuous range of values without obvious gaps, while the chromatin accessibility state of a gene manifests binary values. These distinct properties of data invalidated techniques capturing linear associations such as correlation coefficients or linear regressions. We captured the strength of those non-linear associations by dividing tissues into highly mutated and lowly/non-mutated groups and evaluating the difference between the median feature values of the two groups. Statistical significance was assessed by randomly permuting the feature values among tissues. This measure can also determine the direction of associations: positive if the mean feature value difference between highly and lowly/no-mutated groups is positive and negative if the difference is negative. Fig. S5 shows examples of positive (top), negative (bottom) and no (middle) associations. Detailed procedures of evaluating association strength and significance are described in Materials and Methods.

Table 1 reports the number and fraction of genes whose mutational profiles are significantly associated with each candidate feature (permutation p-value < 0.03), and their estimated false discovery rates (FDR) respectively. The coverage of associations, gauged by the fraction of genes with significant associations, had the following order: pathway activity (33.6%, 40 of 119 genes, estimated FDR 30.36%), gene expression (22.7%, 98 of 432 genes, estimated FDR 29.54%), protein-protein binding partner activity (19.7%, 50 of 254 genes, estimated FDR 31.58%), and chromatin state (12.1%, 11 of 91 genes, estimated FDR 52.71%). All those features were considerably superior to the control feature of remotely connected gene expression in terms of fraction of significant genes and estimated false discovery rate (9.55% with standard deviation of 1.90%, 24.0 of 251 genes on average, with estimated FDR 67.87%).

**Table 1:** The fraction of genes significantly associated with features tested.

Feature	Total Number of Genes	Number of Significant Genes	Fraction of Significant Genes	Estimated False Discovery Rate
Chromatin State	91	11	0.1209	0.5271
Expression	432	98	0.2269	0.2954
Pathway	119	40	0.3361	0.3036
Protein-protein interaction	254	50	0.1969	0.3158
Control Feature	251	23.9700 ± 4.7810	0.0955 ± 0.0190	0.6787

The table shows for each feature tested, the fraction of significantly associated genes. The total number of genes for each feature depends on data availability. For example, not all genes with expression data have pathway information. The low number of genes for chromatin state is due to the prevalence of genes with constantly EpiON or EpiOFF states across cancer types.

Table S1 reports the fraction of genes with significantly positive associations for each candidate feature. All features were biased towards positive associations, with fractions of genes having significant positive association with chromatin state, gene expression, pathway activity, and protein-protein binding partner activity reported at 72.7%, 87.8%, 92.5%, and 94.0%, respectively. All but one feature (chromatin state) have the positive association bias near or beyond the confidence interval derived from the control feature (58.5% ± 30.1%, two standard deviations from the mean) (for detailed list of significant genes for each feature, please see Table S2). We further extracted known oncogenes and tumor suppressors from positively and negatively associated genes respectively and reported the counts in Table S3. A naïve anticipation is that oncogenes have elevated activities in the functioning tumor types, and thus have relatively lower activities in the corresponding normal tissues and exhibit negative associations between mutation frequencies and normal tissue activities; while tumor suppressors possess positive associations for the reciprocal argument. On the contrary, this anticipated enrichment pattern was not observed. More complicated relations therefore exist between oncological functions of genes and their expressions in normal tissues.

### Genes with significant associations between the mutational profiles and the pathway activities are enriched with cancer genes

Functional enrichment of significantly associated genes were performed in order to elucidate their potential biological roles. For each feature, positively and negatively associated genes were enriched with distinct biological functions (Table S4). For example, genes positively associated with expression were functionally enriched in nucleotide binding, while negatively associated genes were involved in hormone receptor binding. Interestingly, genes within each feature were associated with cancer-related functions or pathways such as CTCF pathway (chromatin state), cell cycle regulation (gene expression), and cell differentiation (protein-protein interaction), while pathways for cancer, such as “KEGG pathways in cancer”, “KEGG endometrial cancer”, and “KEGG prostate cancer”, were directly implicated when using pathway as a feature for association, suggesting potential role of these features in cancer formation.

**Figure 2:**
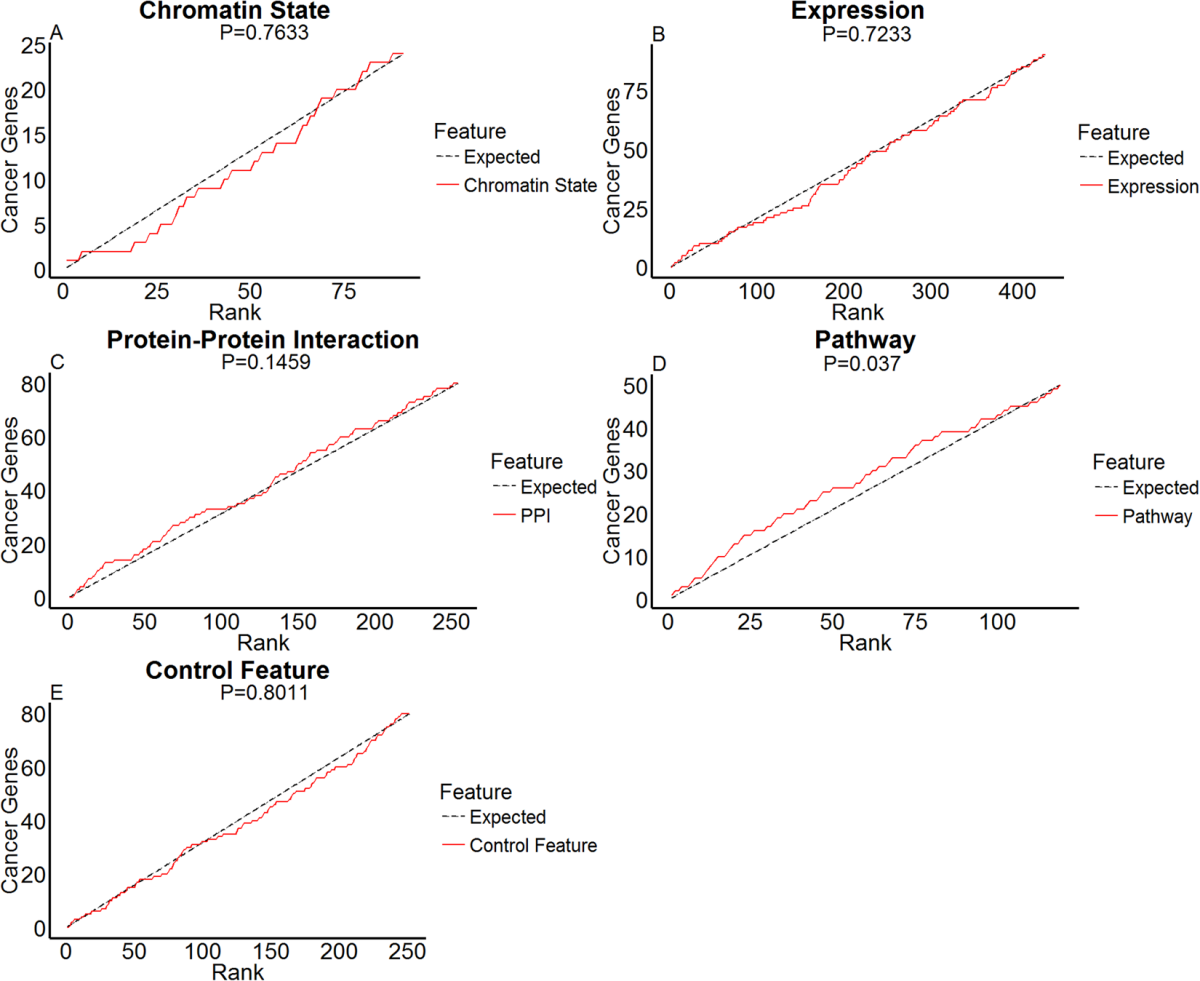
Univariate cancer gene enrichment analysis outcomes. Random walks of gene set enrichments for chromatin state (**A**), expression of the mutated genes (**B**), expression of protein-protein interaction partners (**C**), expression of pathway neighbors (**D**), and the control feature (**E**). In each panel, the *x* axis indicates the rank of a gene in terms of the association significance between its mutation frequencies and feature values, the *y* axis indicates the number of cancer genes within rank *x*. Red solid curves indicate enrichment random walks for the selected features, and black dashed curves indicate the expected random walks when cancer genes are uniformly distributed along the ranks.

To validate biological relevance of normal tissue features in oncogenesis, we employed gene set enrichment analysis (GSEA^61^, and Materials and Methods) and checked whether genes possessing strong associations were enriched with known cancer genes. For each feature, we sorted genes ascendingly according to their association p-values and performed a random walk along the sorted list, with increment +1 when hitting a cancer gene, and 0 otherwise. Figure 2 displays the random walk displacements of the four candidate features and the control feature. For an irrelevant feature, cancer genes are uniformly and randomly distributed along the ranks, thus the displacements approximate a straight line. In contrast, if genes possessing significant associations of a feature are enriched with cancer genes, then cancer genes are concentrated at top ranks and the displacements are positively deviated from the straight line.

Among the four candidate features, the inferred pathway activity has significant enrichment with cancer genes (p-value 0.037). In contrast, associations with expression, inferred protein-protein interaction partner activity, and chromatin states are moderate or poor indicators for cancer genes (p-values 0.1183, 0.1459, and 0.7633 respectively). The control feature random walk exhibits random deviations from the straight line as expected (p-value 0.8011).

When multiple features are informative indicators for a gene set, their influences on enrichment can exhibit a variety of combinatorial scenarios. The enrichment outcomes of some features may be largely contained in other features, relatively independent, or possess epistatic interactions. To capture these dependencies, we developed a novel statistical method to extend GSEA to two features jointly. In brief, we generated three enrichment random walks in addition to the random walks of single features: a joint random walk by counting the union of gene set members falling within each rank in terms of the two features; a conditional random walk by counting the union of expected gene set enrichment between feature 1 and randomly permuted feature 2; and a reciprocal random walk of permuted feature 1 conditioned on feature 2 enrichment outcomes. Feature 2 possesses additional information of gene set enrichment conditioned on feature 1, if the joint random walk is positively deviated from the expected random walk conditioned on feature 1. Conversely, if either the information of feature 2 is contained in feature 1 or feature 2 is not informative at all, then the joint random walk is not positively deviated from the conditional random walk. The detailed procedures of bivariate GSEA are reported in Materials and Methods.

**Figure 3:**
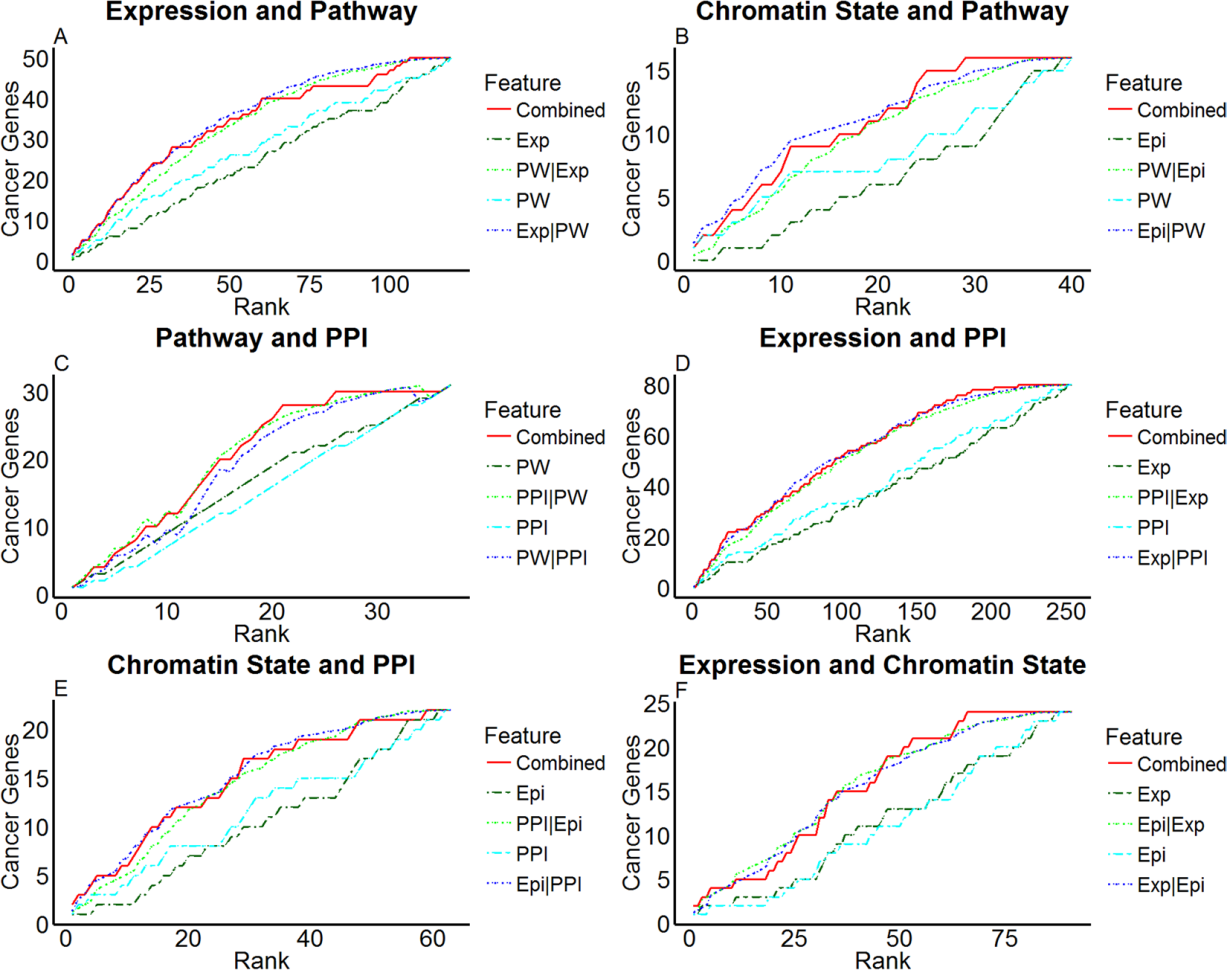
Bivariate cancer gene enrichment analysis outcomes. Each panel shows cancer gene enrichment when taking two features into consideration. For features F1 and F2, F1 and F2 lines represent observed cancer gene enrichment for the respective features, F1|F2 line represents expected enrichment of randomly permuting F1 ranks conditioned on the empirical ranks of F2 (see text), while the “Combined” line represents the observed enrichment contributed by both features. Expression, Chromatin State, Pathway and Protein-Protein Interaction are abbreviated as “Exp”, “Epi”, “PW”, “PPI”, respectively.

We performed bivariate GSEA for each pair of the four features and reported enrichment outcomes in Fig. 3. Pathway activity contained superior information of cancer gene enrichment to expression, as the joint random walk (combined, red) was positively deviated from the expected random walk of pathway activity conditioned on expression (PW|Exp, bright green) but not higher than the expected random walk of expression conditioned on pathway activity (Exp|PW, dark blue) (Fig. 3A). Likewise, pathway activity was superior to chromatin state (Fig. 3B) and protein-protein interaction partner activity (Fig. 3C). Protein-protein interaction partner activity was superior to expression (Fig. 3D) and chromatin state (Fig. 3E). Neither expression nor chromatin state was superior to the other (Fig. 3F). These comparison results are compatible with the univariate GSEA outcomes (Fig. 2) that pathway activity and protein-protein interaction partner activity were the first and second informative features about cancer genes, while expression and chromatin state were not informative about cancer genes.

## Discussion

Among the four candidate normal tissue features analyzed, pathway activity is the most superior in fitting the differential mutations across cancer types (Table 1) and accommodating known cancer genes in gene set enrichment analysis (Figs 2 and 3). It is paradoxical in the first sight that a gene’s neighbors carry more information than itself. Yet a closer look justifies the superiority of pathway activity for at least two reasons. First, as most genes act in a concerted manner, information about activities or transitions of a gene is likely distributed among the network of functions and interactions. Second, from a statistical point of view, average expression data over the measurements of multiple genes is likely more robust than the measurement of a single gene.

The chromatin state of a gene was a strong necessary but insufficient condition for its mutations from tissue-specific study (Fig. 1). However, it also poorly fit differential mutations across cancer types (Table 1) and significantly associated genes were not enriched with known cancer genes (Figs 2 and 3). Furthermore, conflicting evidence of associations between mutation frequencies with closed^62,63^ and open^64,65^ chromatins was previously reported. These incoherent observations suggest the complex and partially known mechanisms relating chromatin states with subsequent mutations. Specifically, two factors may partially account for the disparity in our analysis. First, a necessary but insufficient condition may yield poor association scores since the model of assessing association strength assumes that an open chromatin state is a necessary and sufficient condition for mutation. Second, tissue-specific and cross-tissue analyses considered very disparate sets of genes. A gene can be uniformly accessible (EpiAllON), uniformly inaccessible (EpiAllOFF), or have varying chromatin states (EpiDIFF) across tissue types. Table S5 reports the counts of genes belonging to each epigenomic category in within-tissue and across-tissue studies. In tissue-specific study, EpiAllON and EpiAllOFF genes far exceed EpiDIFF genes. In cross-tissue study, only EpiDIFF genes were selected. The strong contrast between mutation frequency distributions of the two groups is largely attributed to genes with constant chromatin states, which were excluded in cross-tissue study. By restricting to genes with varying chromatin states, the contrast between mutational frequency distributions of the two groups for most cancers (except BRCA, DLBC, and LAML) persists but dwindles (Fig. S6).

The actual cause of associations between mutation frequencies and normal tissue features remains unknown. We suspect that it can be understood in light of cancer genome evolution. Recurrent mutations of a specific gene in a particular cancer type likely imply a strong selection for subclones carrying the mutations, thus substantiate the mutated gene’s important role in the genesis and progression of tumor. Two possible transitions from normal tissues to tumors may account for this outcome. The gene and its contextual partners can be active in the corresponding normal tissue (relative to other tissue types). Its mutation enhances or impairs the pathway activity and thus facilitates tumorigenesis and progression. Alternatively, the gene and its contextual partners can be inactive in the corresponding normal tissue. Mutations awake its activity in cancer and confer a selective advantage in tumor genome evolution. The former and the latter lead to positive and negative associations respectively. Our data suggests that for differential features across normal tissues, the former may be more common than the latter (Table S1).

Non-parametric tests of equality of two distributions such as Kolmogorov-Smirnov (KS) and Mann-Whitney (MW) tests assume that data points are independently drawn from the distributions. This assumption is violated in our analysis (Figs 1–3) due to the correlated data structure. Neighboring genes on the genome can be co-mutated or possess similar chromatin marks, and co-regulated genes can be co-expressed. The statistical significance of our analysis can thereby be overestimated. The overestimation is yet mitigated by two properties of our analysis. First, in Figs 1–3 we treated the p-values of the KS or MW tests as test statistics and employed permutation tests to obtain their p-values. The permutation p-values are less sensitive to sample sizes and correlated data points than KS or MW test p-values. Second, in the original GSEA, the scores of genes were derived by associating their feature values (e.g., expression levels) with a common phenotype (e.g., cancerous/normal tissue labels of samples). Spurious enrichment becomes salient when highly correlated gene expressions are all correlated with erroneous phenotype labels. In our analysis, rather than a common phenotype each gene possesses its own phenotype (mutational profile). The outcomes are thus much less affected by associations between correlated gene features and the same number of noisy phenotype labels.

Both Roadmap and Body Map data were derived from a small number of cancer-free subjects (one person for each tissue, please also refer to Table S6A,B). For Roadmap data, some tissues, such as brain, has multiple measurements from distinct brain regions and these measurements were averaged. The small sample sizes likely reduce the stability of features in normal tissues. The limitation of data cannot be resolved by the present study. Yet the prominent association outcomes seem to surpass this limitation despite the small sample sizes.

Despite the encouraging outcomes, our analysis is too early to accurately predict the occurrences of gene mutations in cancer from normal tissue features. The outcome variables (mutation frequencies) were aggregate statistics over hundreds of cancer patients, and covariates (candidate features in normal tissues) were derived from subjects totally unrelated to cancer patients. A clinically relevant predictor should target characteristics specific to patients and utilize information from the same patients in prediction. For instance, one may predict the occurrence of a particular gene mutation in a specific patient, based on gene expressions of the patient’s normal tissues prior to tumor onset. This would require prospective investigations to collect pre-cancer normal tissues of the patients. However, it was not our intention to build such predictors, as many efforts have been dedicated to finding biomarkers for diagnosis and prognosis of cancers. Rather, our study can be viewed as an early step toward understanding molecular and clinical characteristics of cancers from an evolutionary perspective. Many recurrent patterns in cancers – such as population-specific mutations, progression stage-specific mutations, and tissue-specific metastasis – are richly documented but remain poorly explained. Investigation from an evolutionary perspective, such as examining the functions and activities of genes in normal tissues, will likely shed light on those problems. Deeper knowledge about the relation between cancer genome evolution and these patterns will facilitate the prevention, diagnosis and treatment of cancers and have tremendous impacts on human health.

## Materials and Methods

### Data Collection and Processing

Somatic mutation data of 19 tumor types called by MuTect algorithm was downloaded from the TCGA data portal^66^ (https://tcga-data.nci.nih.gov/tcga/, currently hosted at The Genomics Data Commons Data Portal https://gdc-portal.nci.nih.gov/). For protein-coding genes, only nonsynonymous mutations were included in the calculation of mutation frequency, as they altered protein sequences and thus were more likely to change protein functions. Mutation frequency of each protein coding gene in each tissue was counted as the fraction of patients harboring any nonsynonymous mutation on that gene. For non-protein coding genes, such as microRNAs, all sequence mutations were included in the calculation of mutation frequency. Abbreviations for cancer types follows the notations used by TCGA (https://gdc.cancer.gov/resources-tcga-users/tcga-code-tables/tcga-study-abbreviations).

Epigenomics data of normal tissues was downloaded from the Roadmap Epigenomics Project^67^ (http://egg2.wustl.edu/roadmap/web_portal/). The data contained predictions regarding genome-wide chromatin states in individual tissues based on an integrated model of 127 epigenomes with 25 epigenomics marks (such as H3K27me3 and H2A.Z). For a tissue, the data (downloaded as genomic coordinates) were converted to gene symbols using biomaRt^68^. Genes with states “Active Transcription”, “Transcribed – 5’ preferential”, “Strong transcription” and “Transcribed – 3’ preferential” were subsequently labeled as accessible for activate transcription and the remaining were labeled as inaccessible (henceforth as “EpiON” and “EpiOFF”, respectively). As with gene expression data, normal tissues were mapped to the corresponding tumor types based on the same guidelines (Table S6). 11 out of 19 tumor types has corresponding epigenomic data for normal tissues. If a tumor type has more than one corresponding epigenomic data for normal tissues, for a particular gene, more than half of the data has to be “EpiON” for the gene to be considered as “EpiON”.

Illumina Body Map, the RNA-Seq gene expression data of normal human body tissues used in this study, was downloaded from EBI Expression Atlas^69^ (https://www.ebi.ac.uk/gxa/home). Data from 15 normal tissues types were mapped to 19 corresponding tumor types studied by the TCGA project (Table S6), primarily based on TCGA Enrollment Form (http://www.nationwidechildrens.org/tcga-clinical-data-forms-standard) “Primary Site of Disease” and “Histological Subtype” sections. If a tissue has multiple tumor types, all of them are considered individually, for example, gene expression data for kidney tissue was used for all 3 kidney tumor types, KIRC, KICH and KIRP. Raw FPKM values were Z-normalized across before further study.

Human biological pathway data was downloaded from KEGG^70^ (http://www.genome.jp/kegg/kegg2.html). Human protein-protein interaction data was downloaded from Human Protein Research Database (HPRD)^71^ (http://www.hprd.org/). List of cancer genes was downloaded from COSMIC Cancer Gene Census^17^ (http://cancer.sanger.ac.uk/census/).

### Assessing statistical significance of the score deviation between two groups of genes

For each tissue type, we subdivided genes into EpiON and EpiOFF groups according to their chromatin states, and displayed their frequency mutation distributions in Fig. 1. To assess the statistical significance of the deviation between EpiON and EpiOFF group distributions, we calculated the p-values of one-sided KS tests (assuming that the EpiON group yielded higher mutation frequencies) and used them as the test statistics. We then randomly permuted the chromatin state labels of genes 100,000 times and reported the p-value as the fraction of random permutations whose KS p-values were below the one from empirical data. To demonstrate that permutation p-values were robust against sample sizes, we subsampled genes by 5, 10, 25, 50, and 100 folds and reported their mutation frequency distributions and permutation p-values in Figs S7–S11. One-sided KS tests were employed based on the assumption that genes with an open chromatin were more prone for subsequent mutations. This assumption was validated by showing that the outcomes derived from two-sided KS tests were less significant than Fig. 1 (Table S7) and the outcomes derived from one-sided KS tests of the opposite direction were insignificant (Table S7). Figure S1 was likewise generated by replacing mutation frequencies with gene expressions.

We also subdivided genes into highly expressed and lowly expressed groups and visualized their mutation frequency distributions. Unlike chromatin states, the expression level of a gene is a continuous value rather than a discrete state. We thus varied the threshold value separating the two groups to FPKM > 10, 50 percentiles and 75 percentiles and displayed their distributions in Figs S2–S4. The difference of mutation frequency distributions between the two groups remained low among all three threshold values.

### Pathway, PPI, and gene expression feature value calculation

We quantified the pathway activity of a gene in a specific tissue with the following procedures. First, we selected all the first-degree neighbors of the designated gene in the pathway and calculated their mean expression value. Second, we randomly drew the same number of genes 100,000 times and calculated the mean expression values in all random trials. Third, the p-value of the pathway neighbors’ mean expression value was the fraction of random trials whose mean expression values exceeded the empirical value. Negative log of the p-value was then used as the pathway activity. Similar calculations were performed for gene expression and the PPI network by considering only the gene’s expression value, and first-degree neighbors in the network, respectively.

### Testing the association between variations of mutation frequency and a feature in normal tissues

We devised a permutation test to quantify the association significance between each candidate feature and mutation frequency.

1. For chromatin state, we split tissues into the groups with accessible chromatins (“EpiON” group) and inaccessible chromatins (“EpiOFF” group). The test statistic was the difference of median mutation frequencies between EpiON and EpiOFF groups.
2. For other features, we subdivided tissues according to their mutation frequencies instead, as a gene was often highly mutated in only a few tissues. We sorted tissues according to their mutation frequencies ascendingly. Successive data point pairs with the largest gap of mutation frequencies were chosen as the boundary point to split tissues. Data points below the low boundary point (including the low boundary point) were assigned to the lowly mutated group 1. Data points above the high boundary point (including the high boundary point) were assigned to the highly mutated group 2. The test statistic was the difference of median feature values between group 2 and group 1.
3. We randomly permuted mutation frequencies of tissues 100,000 times and evaluated the test statistic in each random trial. The p-value was the fraction of random test statistic values exceeding the empirical test statistic value.
4. When a gene was mapped to multiple pathways, we set the pathway activity p-value as the minimum of p-values over the mapped pathways. This criterion selected the strongest pathway associated with mutational profiles rather than requiring all mapped pathways to be strongly associated with mutational profiles.
5. The false discovery rate of a feature was estimated by comparing the number of significant associations in the empirical data and the number of significant associations in the randomized data. For each gene we permuted its mutation frequency labels 100 times. Steps 1–4 were applied to the permuted data to obtain (false positive) significant associations. FDR was calculated as

$$False\,Discovery\,Rate=\frac{\#{\rm{Signficant}}\,{\rm{genes}}\,{\rm{in}}\,{\rm{the}}\,{\rm{permuted}}\,{\rm{data}}}{\#{\rm{Significant}}\,{\rm{genes}}\,{\rm{in}}\,{\rm{the}}\,{\rm{empirical}}\,{\rm{data}}\times 100}$$

### Control feature generation

The control feature of a gene was obtained from its remote neighbors in the protein-protein interaction network. The *n*th-degree neighbors of a target gene are the genes in the protein-protein interaction network whose shortest distances from the target gene are *n*. We counted the numbers of genes possessing at least five neighbors from the first to eighth degrees (Fig. S12). The numbers remain relatively stable between the third to seventh degrees. Therefore, for each gene we randomly selected 5 genes from its third to seventh-degree neighbors in the protein-protein interaction network and used their mean expression value as the control feature. Association p-values of the control feature were evaluated by the aforementioned permutation test as other features. We performed 100 random trials to generate control features and calculated the mean and standard deviation of association coverage in Table 1.

### Functional annotation of significantly associated genes

Genes with association p-value < 0.03 from each category was subjected to functional annotation analysis using an online tool hosted at http://software.broadinstitute.org/gsea/msigdb/annotate.jsp. Gene sets queried included “H: Hallmark gene sets”, “C2: Curated gene sets”, “C5: Gene Ontology gene sets”, “C6: Oncogenic signatures”, and “C7: Immunologic signatures”.

### Univariate gene set enrichment analysis

To test whether the association between the mutational profile and a candidate feature was informative about the cancer processes, we adopted gene set enrichment analysis as previously proposed^61^.

1. As the basis of all association studies, we selected 432 differentially mutated genes by taking the union of the top-50 frequently mutated genes in each of the 19 cancer types in TCGA.
2. Association p-values between mutational profiles and each candidate feature were evaluated for the selected genes. The selected genes were the intersection of the 432 basis genes and genes possessing each feature: 432 genes with gene expression data, 91 genes with differential chromatin states, 119 genes with mapped pathways, and 254 genes with protein-protein interactions.
3. The gene set considered was the list of 90 cancer genes extracted from the COSMIC database overlapped with selected 432 differentially mutated genes.
4. For each feature, we sorted genes by their association p-values in an ascending order.
5. Define *x* as the rank of genes in terms of association p-values, and *y*(*x*) as the number of cancer genes above/equal to rank *x*. *y*(*x*)was obtained by a random walk along the sorted genes. Starting with 0, *y*(*x*) incremented by 1 if the gene of rank *x* was a cancer gene, and 0 otherwise. The resulting *y*(*x*) was a non-decreasing function.
6. If a feature was informative about cancer processes, then the top-ranking genes – genes with strong association p-values – were anticipated to be enriched with cancer genes. Therefore, the random walk would quickly gain a high value and remain stable subsequently.
7. The null hypothesis is that the feature was uninformative about cancer processes, and whether a cancer gene was included in the top-ranking list was completely determined by random chance. We defined *N* as the total number of genes, and *K* as the total number of cancer genes. At step *n* of a walk, there were *n* top-ranking genes and *k* of them were cancer genes. The probability of randomly choosing *k* cancer genes from the top *n* genes was given by a hypergeometric distribution:
  1 $${P}_{n,k}=P(k\,cancer\,genes\,in\,top\,n\,genes\,|random\,walks)=\frac{(\begin{array}{c}K\\ k\end{array})(\begin{array}{c}N-K\\ n-k\end{array})}{(\begin{array}{c}N\\ n\end{array})}$$
  The expected of the number of cancer genes from the top *n* genes was given by
  2 $$Expected[Number\,of\,cancer\,genes\,in\,top\,n\,genes]=\sum _{k=0}^{min(n,K)}{P}_{n,k}\cdot k\,$$
  This expected number approximated the expected number $\frac{n\cdot K}{N}$ where a cancer gene was randomly drawn with probability $\frac{K}{N}$ and replacement. The random walk of the null model thus approximated a straight line $y(x)=\frac{K}{N}\cdot x$.
8. The significance of cancer gene enrichment was quantified by the positive deviation of the *y*(*x*) obtained by the empirical data from the *y*(*x*) obtained by the null model, which was a straight line for a single feature. Specifically, we normalized random walk curves to 0 ≤ *y*(*x*) ≤ 1 and treated them as cumulative distribution functions (CDFs) of random variables. P-values were calculated by comparing empirical Mann-Whitney U test statistics with corresponding 100,000 permutation test statistics.

### Bivariate gene set enrichment analysis

Classical GSEA assesses whether a feature contains information about a particular gene set. We extended this framework to multiple features and assessed whether multiple features jointly provided extra gene set information relative to subsets of those features. In this study, we considered two features concurrently, although the framework can be easily extended to more features.

1. We first defined the random walk of enrichment *y*(*x*) as for univariate GSEA. Two sorted gene lists were generated according to the association p-values of the two features F1 and F2 respectively. *y*_*F*1*F*2_(*x*) was defined as the number of cancer genes in the union of top *x* genes from the two lists, and *y*_*F*1_(*x*) and *y*_*F*2_(*x*) were the number of cancer genes in each list respectively. Obviously, *y*_*F*1*F*2_(*x*) was not lower than *y*_*F*1_(*x*) and *y*_*F*2_(*x*) for any *x*.
2. To test whether the two features jointly provided more enrichment information than F1 alone, we constructed a null model curve *y*_*F*2|*F*1_(*x*). Conditioned on the enrichment *y*_*F*1_(*x*)of the F1 list at rank *x*, *y*_*F*2|*F*1_(*x*) specified the expected number of cancer genes when F2 was not informative about the gene set. *N* and *K* were defined as univariate GSEA. At rank *n* there were *n* top-ranking genes and *k* cancer genes from the F1 list. Suppose by incorporating a randomly sorted F2 list *n*_*extra*_ genes and *k*_*extra*_ cancer genes were added. The probability that randomly selected *n* genes added *n*_*extra*_ genes to the sorted F1 list of *n* genes was given by a hypergeometric distribution
  3 $$\begin{array}{rcl}{P}_{{n}_{extra}|n} & = & \,P({n}_{extra}\,genes\,contributed\,by\,top\,n\,genes\,in\,F2|F1)\\  & = & \frac{(\begin{array}{c}N-n\\ {n}_{extra}\end{array})(\begin{array}{c}n\\ n-{n}_{extra}\end{array})}{(\begin{array}{c}N\\ n\end{array})}\,\end{array}$$
  Furthermore, conditioned on those *n*_*extra*_ genes, the probability that *k*_*extra*_ of them were cancer genes was given by another hypergeometric distribution
  4 $$\begin{array}{rcl}{P}_{{k}_{extra}|{n}_{extra}} & = & \,P({k}_{extra}\,cancer\,genes\,by\,F2|{n}_{extra}\,genes\,by\,F2)\\  & = & \,\frac{(\begin{array}{c}K-k\\ {k}_{extra}\end{array})(\begin{array}{c}N-n-K+k\\ {n}_{extra}-{k}_{extra}\end{array})}{(\begin{array}{c}N-n\\ {n}_{extra}\end{array})}\,\end{array}$$
  The expected number of extra cancer genes included in the union of the two top-*n* lists then becomes
  5 $$\begin{array}{c}Expected\,[Number\,of\,extra\,cancer\,genes\,contributed\,by\,F2|F1]\\ \,\,=\sum _{{n}_{extra}=0}^{{\rm{\min }}(n,N-n)}\,\sum _{{k}_{extra}=0}^{{\rm{\min }}({n}_{extra},K-k)}{{\rm{P}}}_{{n}_{extra}|n}\cdot {{\rm{P}}}_{{k}_{extra}|{n}_{extra}}\cdot {k}_{extra}\end{array}$$
  which was *y*_*F*2|*F*1_(*n*).
3. Compared the empirical random walk from the joint list *y*_*F*1*F*1_(*x*) and the expected random walk conditioned on the F1 list *y*_*F*2|*F*1_(*x*). If *y*_*F*1*F*2_(*x*) was positively deviated from *y*_*F*2|*F*1_(*x*), then F2 provided additional information about gene set enrichment after F1 was taken into account. The significance was again quantified by the Mann-Whitney permutation p-value. The additional information of F1 relative to F2 was assessed analogously by comparing *y*_*F*1*F*2_(*x*) with *y*_*F*1|*F*2_(*x*).
4. There are four possible outcomes of (*y*_*F*1*F*2_(*x*), *y*_*F*2|*F*1_(*x*)) and (*y*_*F*1*F*2_(*x*), *y*_*F*1|*F*2_(*x*)) comparisons:
  *y*_*F*1*F*2_(*x*) is positively deviated from both *y*_*F*2|*F*1_(*x*) and *y*_*F*1|*F*2_(*x*) – F1 and F2 both provide indispensable enrichment information.
  *y*_*F*1*F*2_(*x*) is positively deviated from *y*_*F*2|*F*1_(*x*) but not *y*_*F*1|*F*2_(*x*) – F2 is superior to F1 in gene set enrichment.
  *y*_*F*1|*F*2_(*x*) is positively deviated from *y*_*F*1|*F*2_(*x*) but not *y*_*F*2|*F*1_(*x*) – F1 is superior to F2 in gene set enrichment.
  *y*_*F*1|*F*2_(*x*) is not positively deviated from either *y*_*F*2|*F*1_(*x*) or *y*_*F*1|*F*2_(*x*) – neither F1 nor F2 is informative in gene set enrichment, or F1 and F2 provide largely overlapped enrichment information.

## Electronic supplementary material

Supplementary File 1
