# Supplementary material for: Explaining cancer type specific mutations with transcriptomic and epigenomic features in normal tissues
Source: Sci Rep. 2018 Jul 30;8:11456. doi: 10.1038/s41598-018-29861-1 (PMC6065413; doi:10.1038/s41598-018-29861-1)
Supplement: Supplementary file 1 [file 41598_2018_29861_MOESM1_ESM.pdf]

Supplementary information for:

Explaining cancer type specific mutations with transcriptomic and epigenomic features in normal tissues

Khong-Loon Tiong[1], Chen-Hsiang Yeang[1*]

[1]*Institute of Statistical Science, Academia Sinica, Taipei, Taiwan*

[*]*Corresponding author:* chyeang@stat.sinica.edu.tw

Supplementary Figures

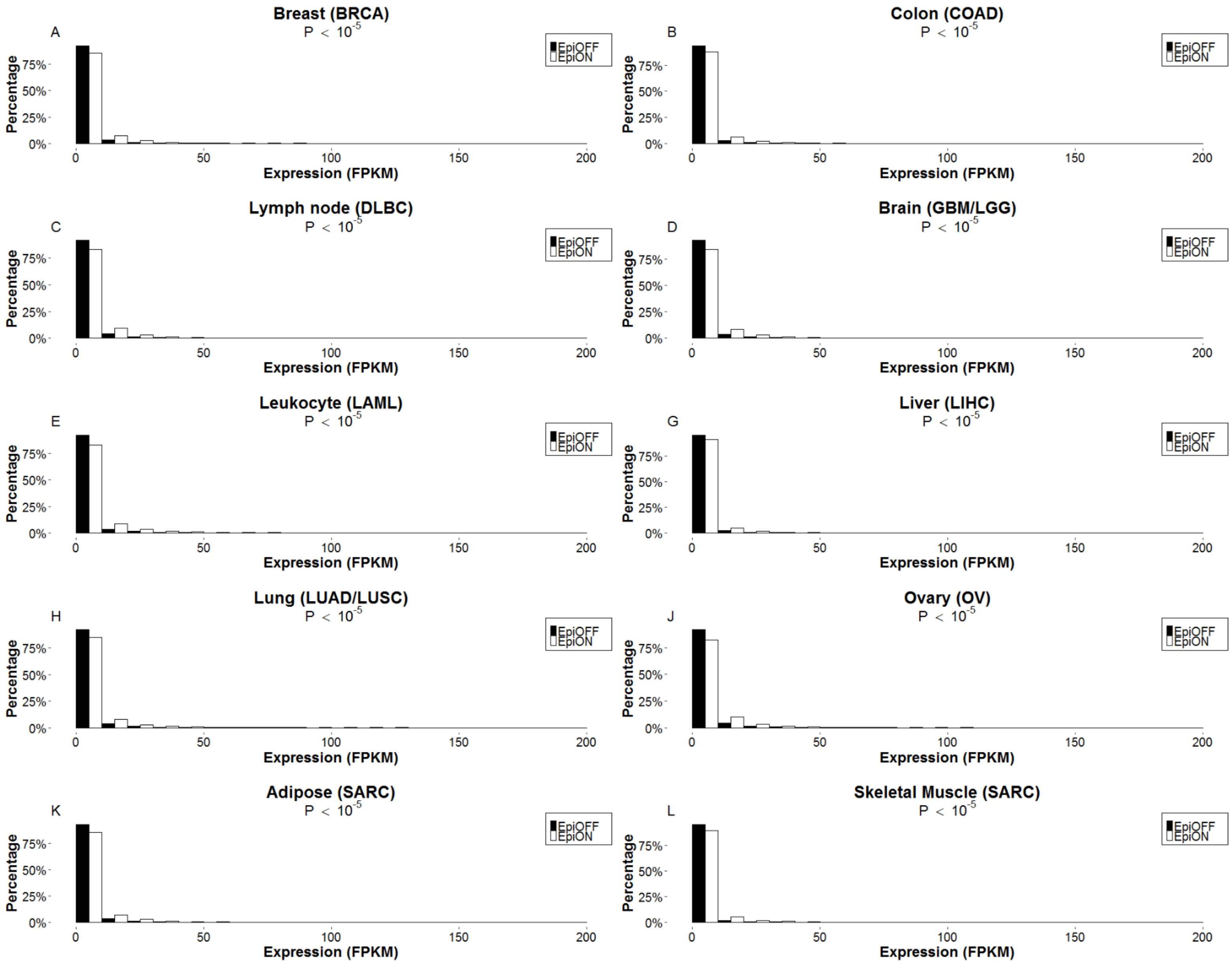

Figure S1. Tissue-specific distribution of expression level of EpiON and EpiOFF genes

Genes were classified into EpiON and EpiOFF genes. Each panel shows the distributions of expression levels (in FPKM) of EpiON (white bars) and EpiOFF (black bars) genes in the corresponding normal tissue of the designated cancer type. The permutation p-value for each tissue type is provided.

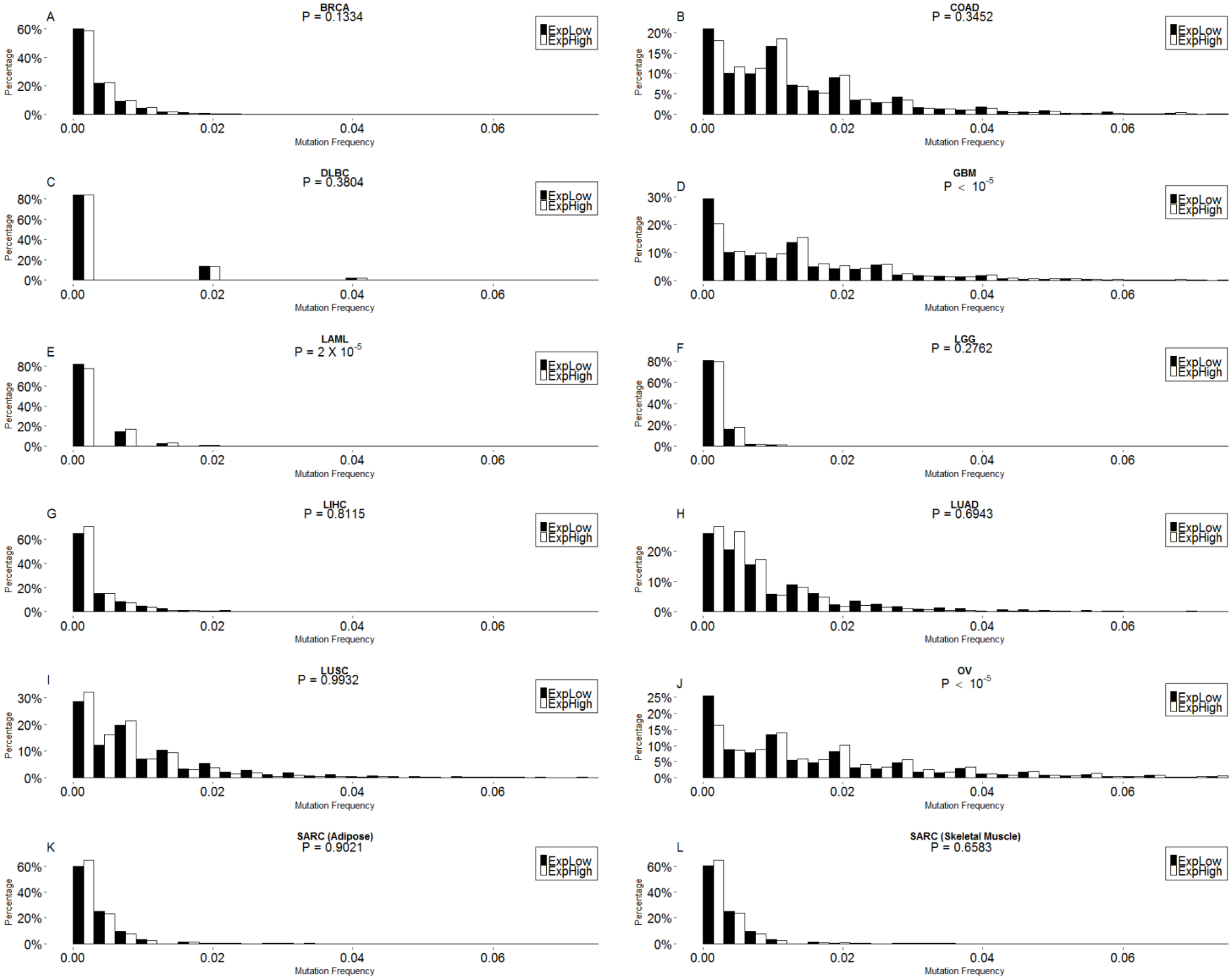

Figure S2. Tissue-specific distributions of mutation frequency of ExpHigh and ExpLow genes using FPKM > 10 as cutoff

Genes were classified as ExpHigh if their gene expression values exceed the cutoff and as ExpLow otherwise. Each panel shows the distributions of mutation frequency of ExpHigh and ExpLow genes in the designated cancer type.

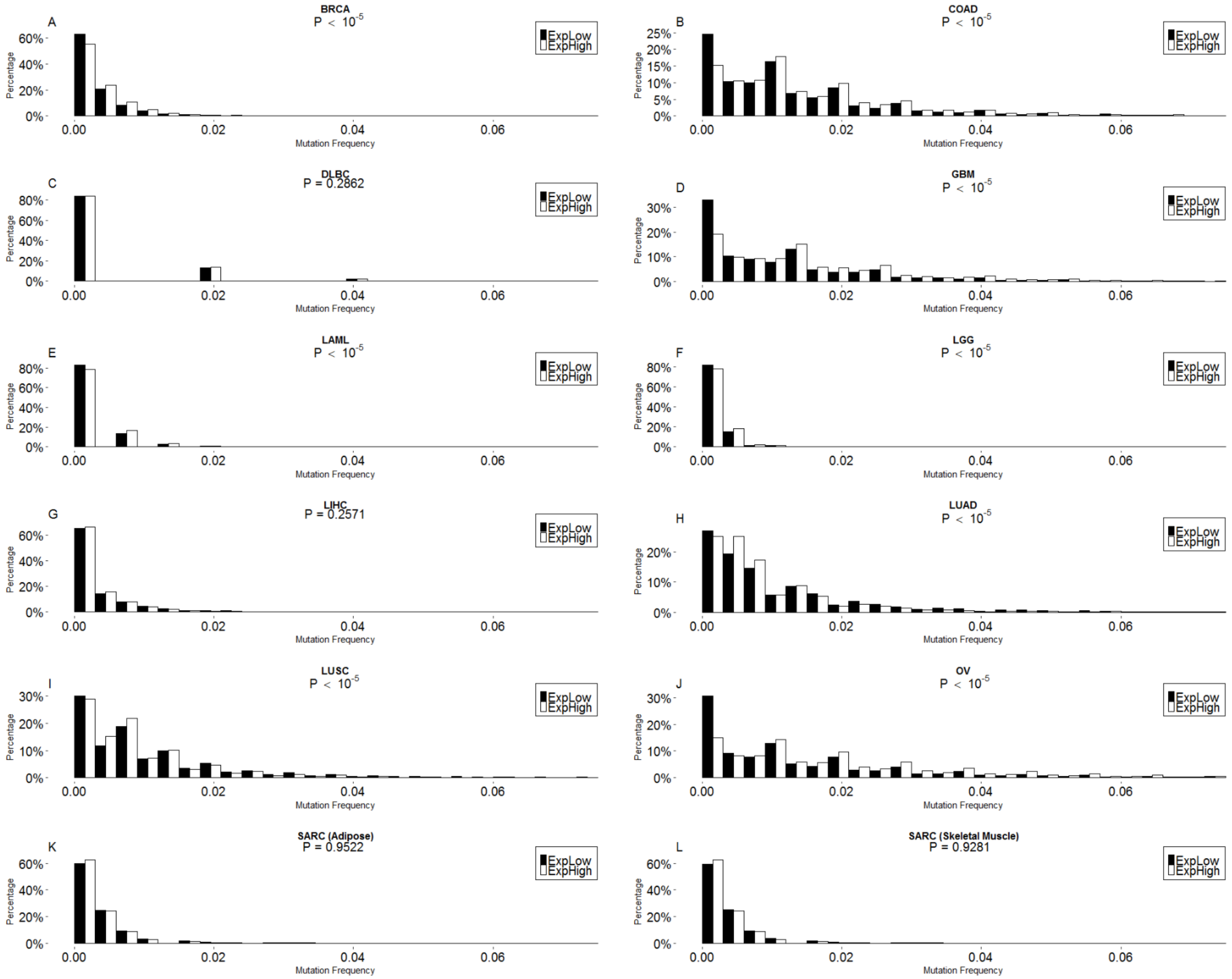

Figure S3. Tissue-specific distributions of mutation frequency of ExpHigh and ExpLow genes using 50 percentiles as cutoff

Legends and captions follow Fig S2.

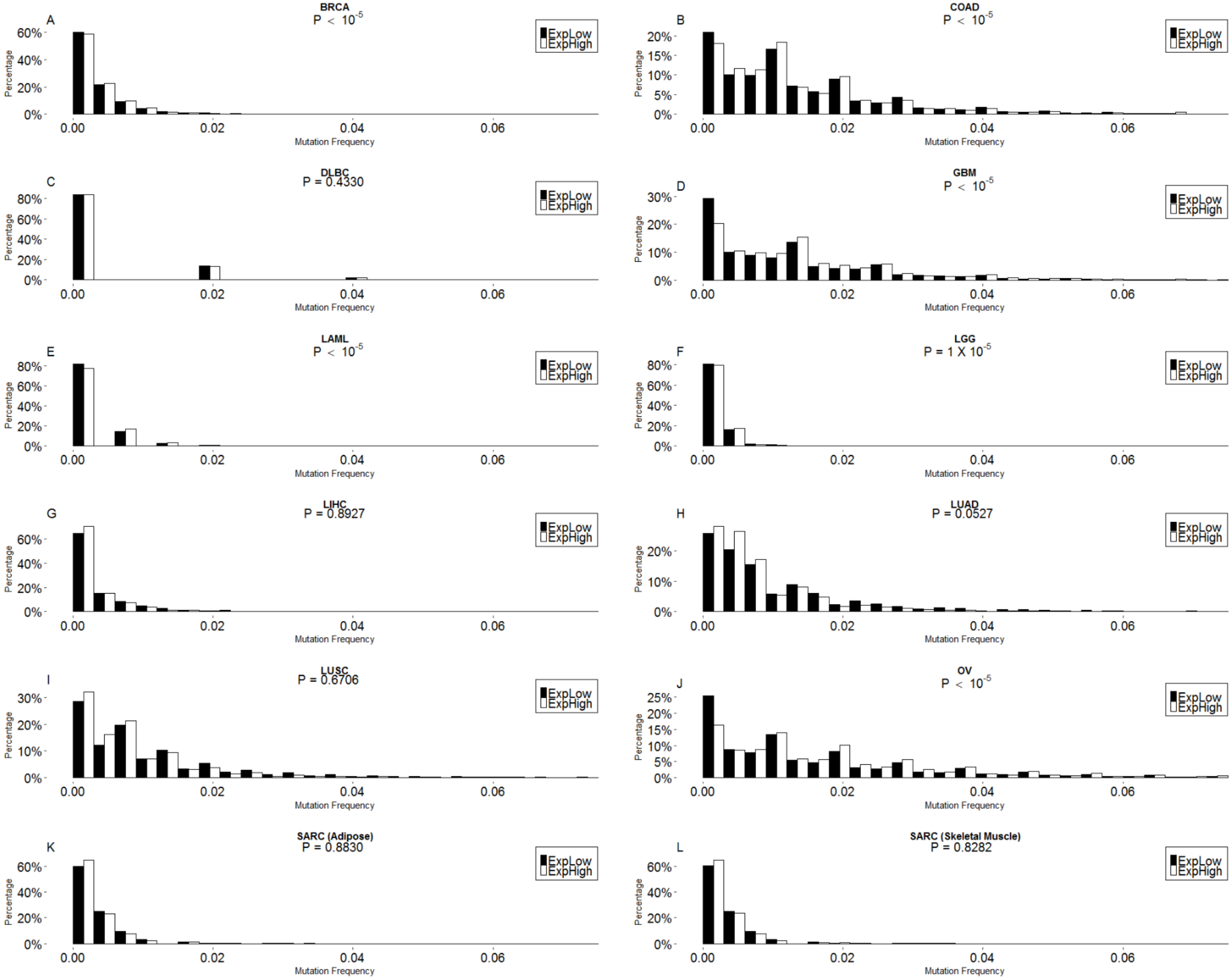

Figure S4. Tissue-specific distributions of mutation frequency of ExpHigh and ExpLow genes using 75 percentiles as cutoff

Legends and captions follow Fig S2.

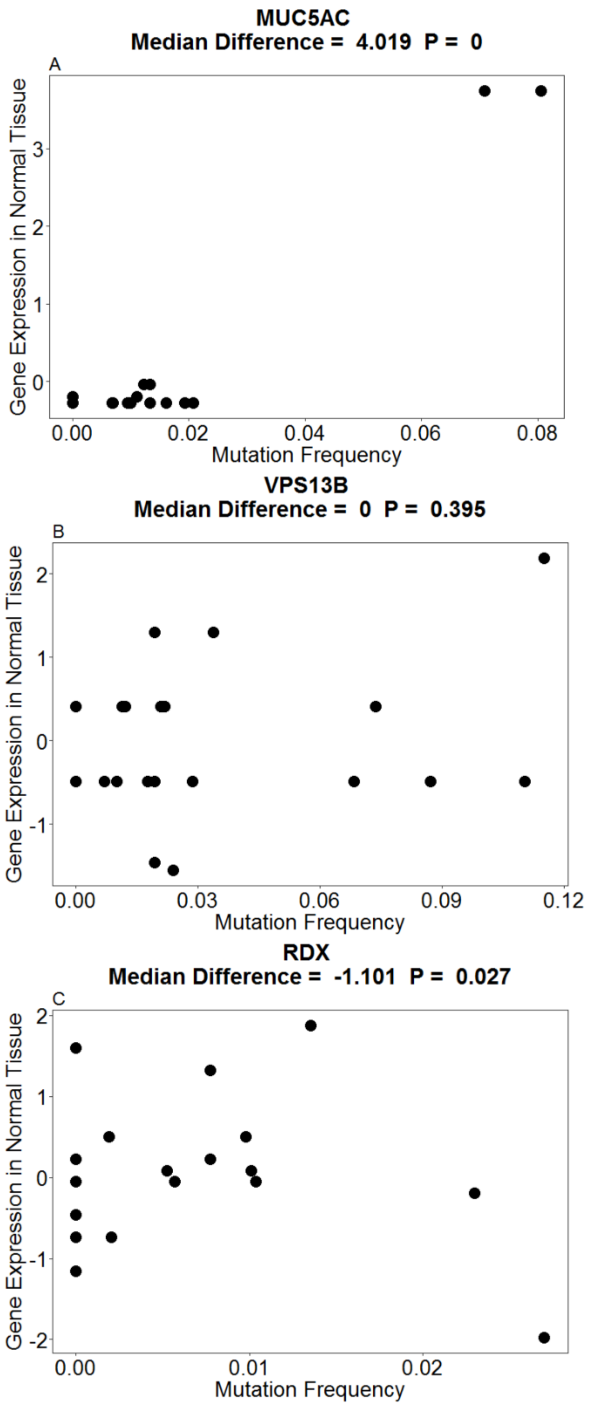

Figure S5. Illustrative examples of directionality of association

Each panel shows a gene with [S5A] significant positive, [S5B] insignificant, and [S5C] significant negative association, between normal tissue gene expression and mutation frequency, respectively.

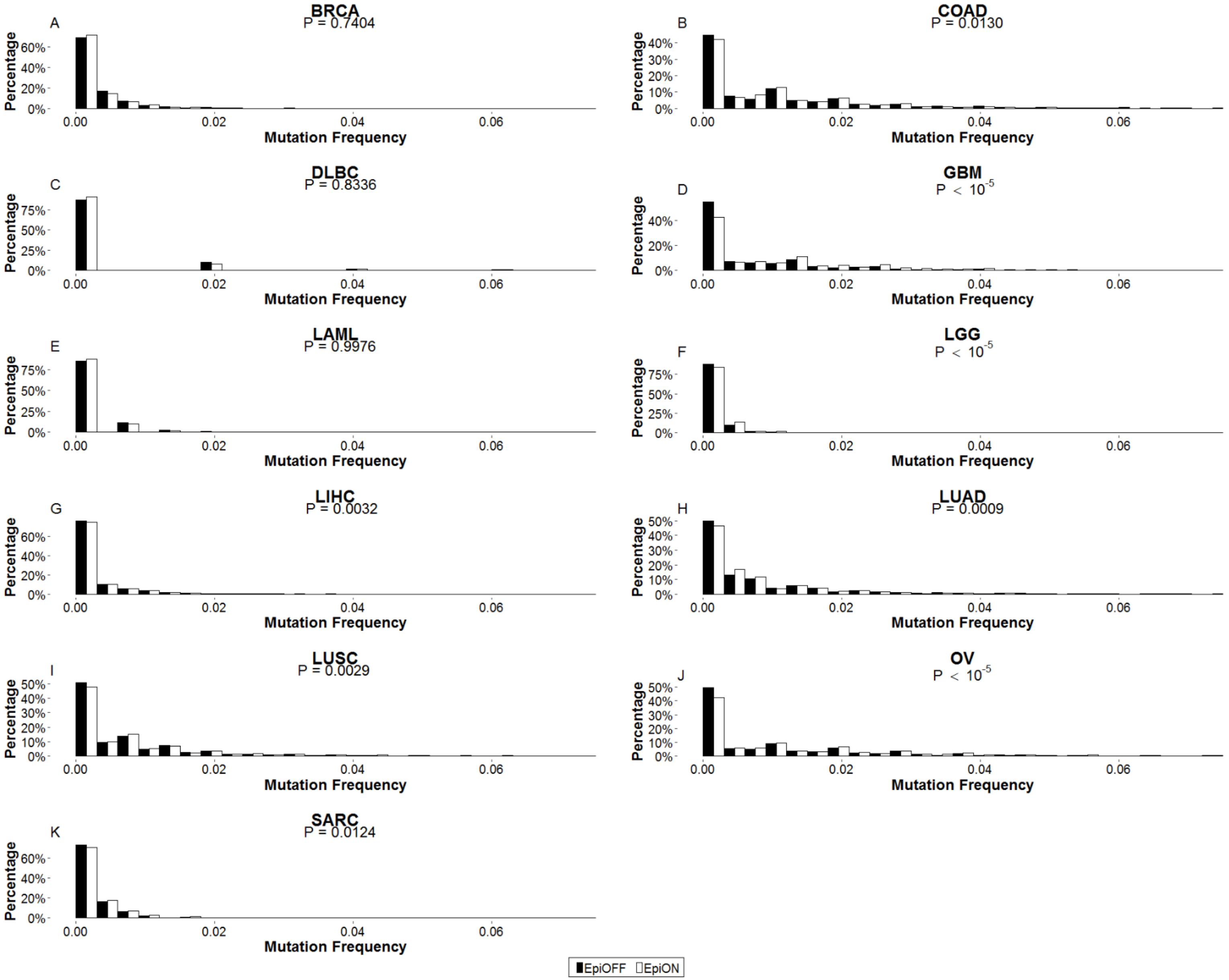

Figure S6. Tissue-specific distributions of mutation frequency of EpiON and EpiOFF genes when considering only genes with different chromatin states across tissues

Legends and captions follow Fig 1.

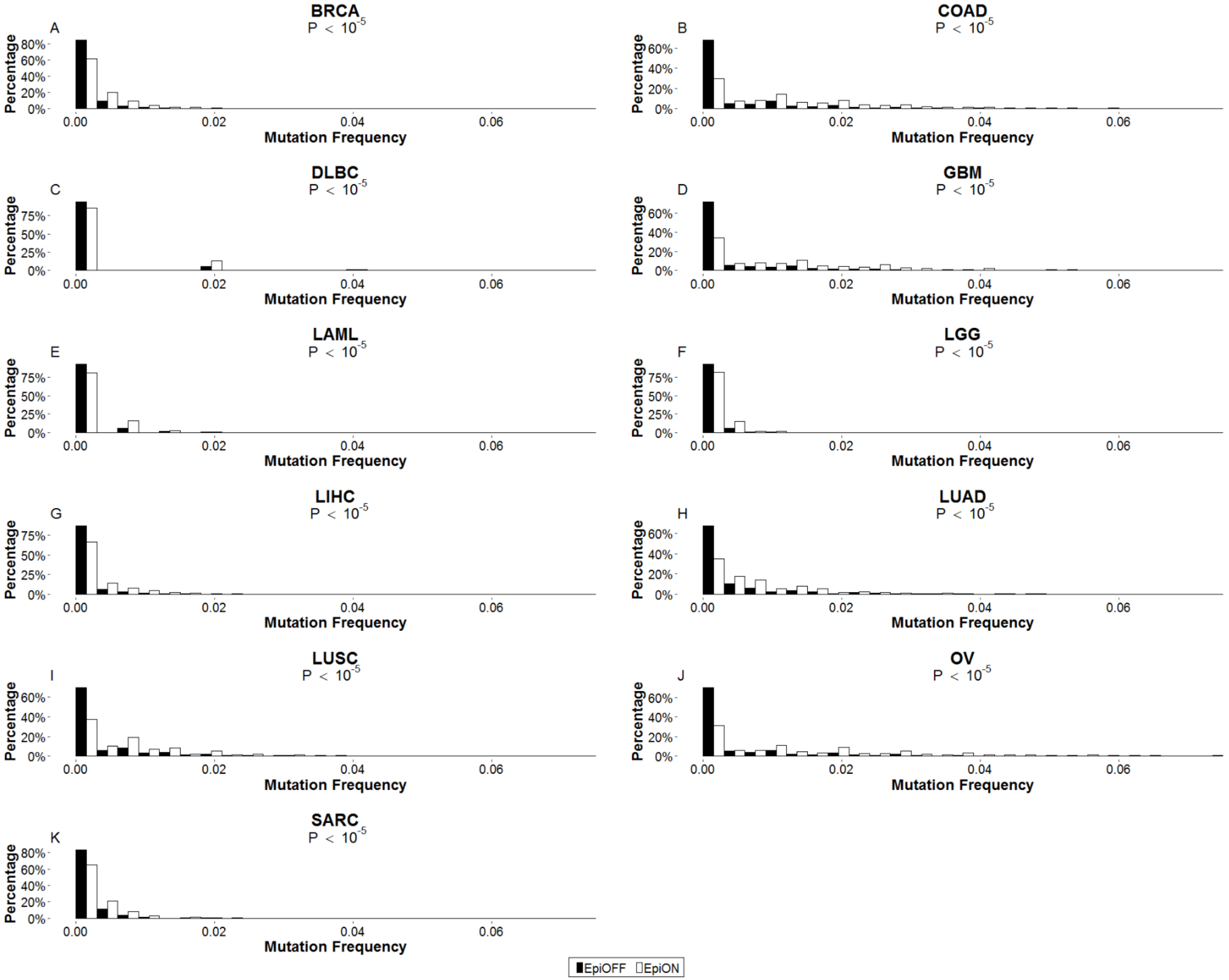

Figure S7. Tissue-specific distributions of mutation frequency of EpiON and EpiOFF genes after 5-fold subsampling

Genes were subsampled by 5-fold before computing the mutation frequency distributions in EpiON and EpiOFF genes. Legends and captions follow Fig 1.

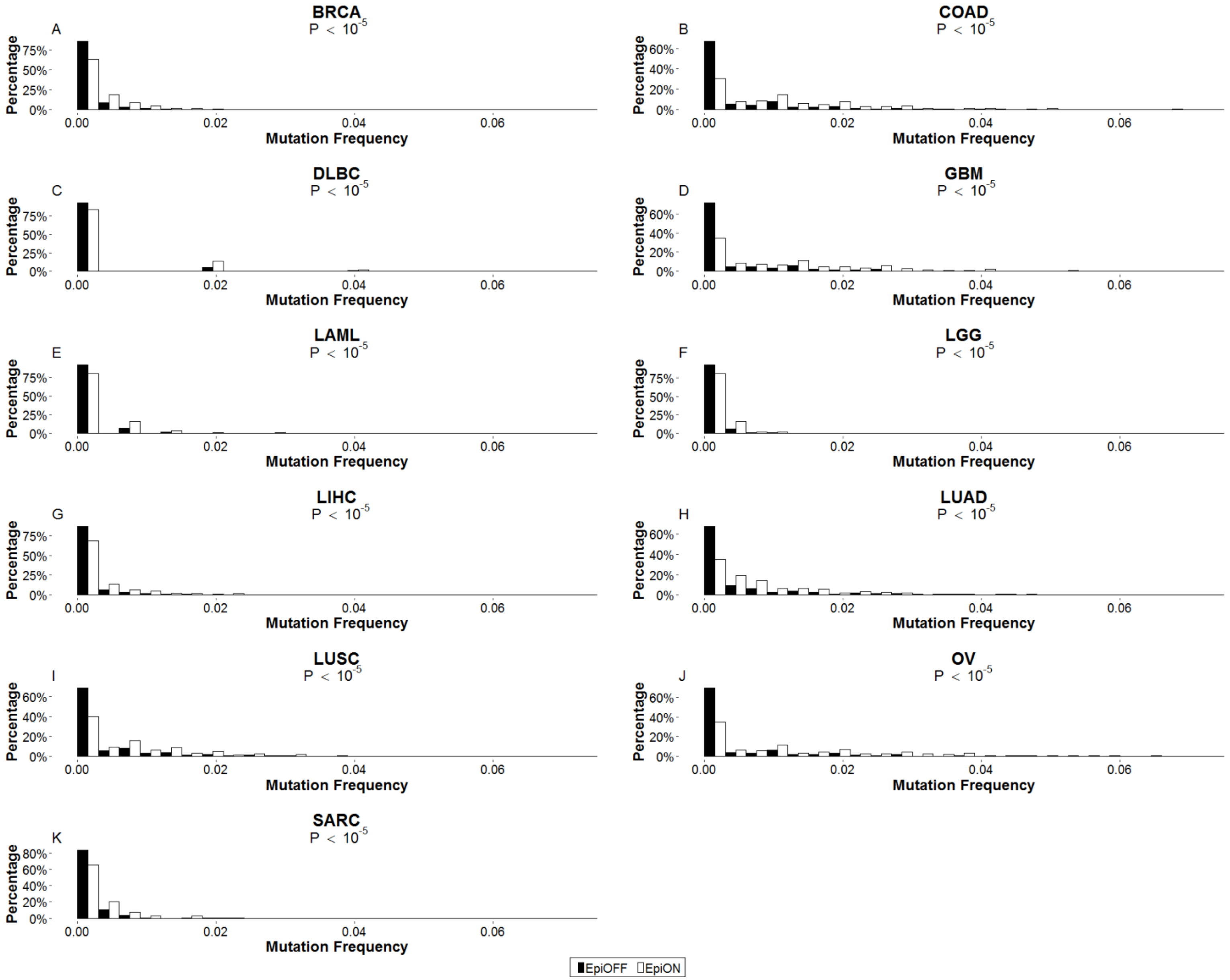

Figure S8. Tissue-specific distributions of mutation frequency of EpiON and EpiOFF genes after 10-fold subsampling

Legends and captions follow Fig 1.

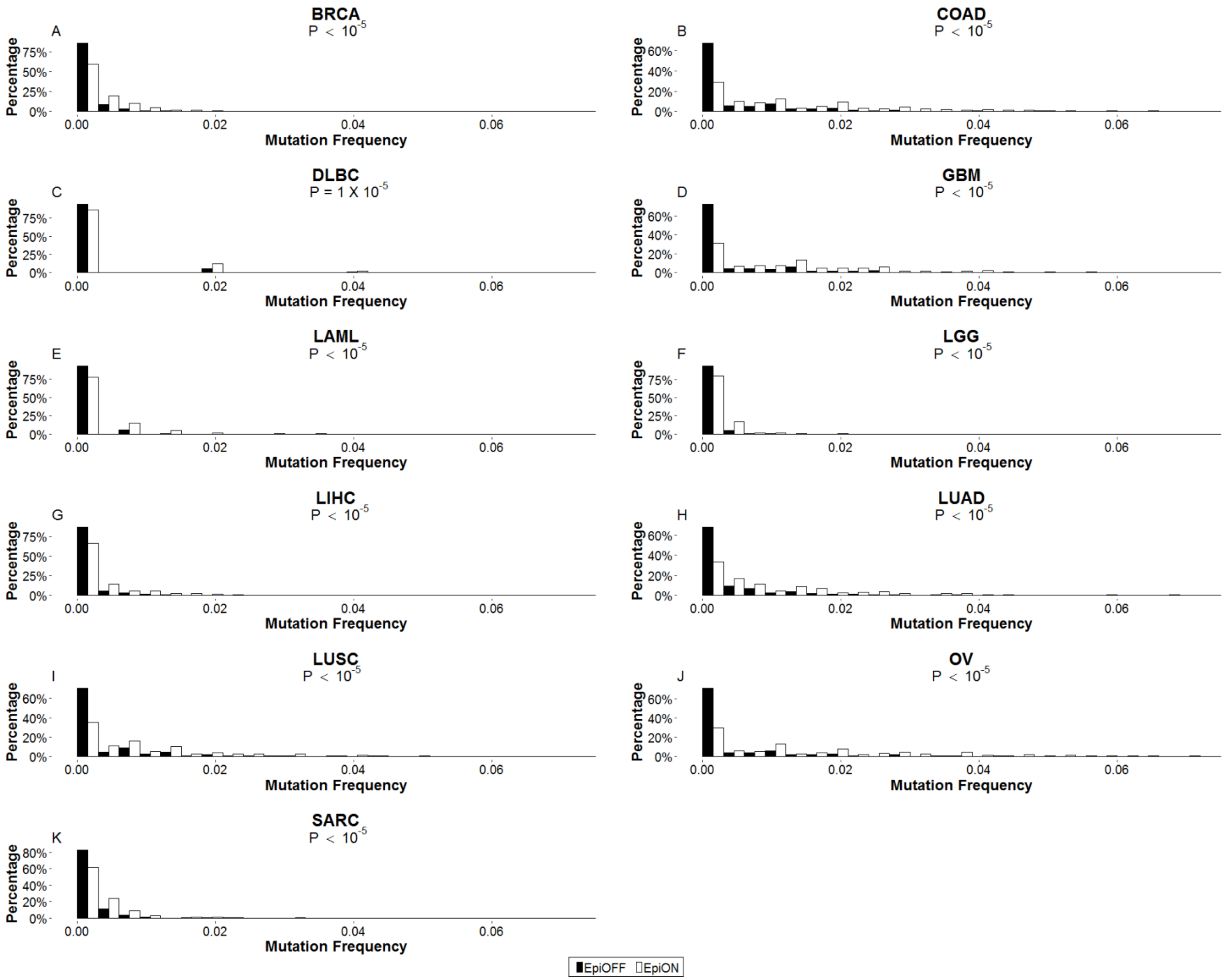

Figure S9. Tissue-specific distributions of mutation frequency of EpiON and EpiOFF genes after 25-fold subsampling

Legends and captions follow Fig 1.

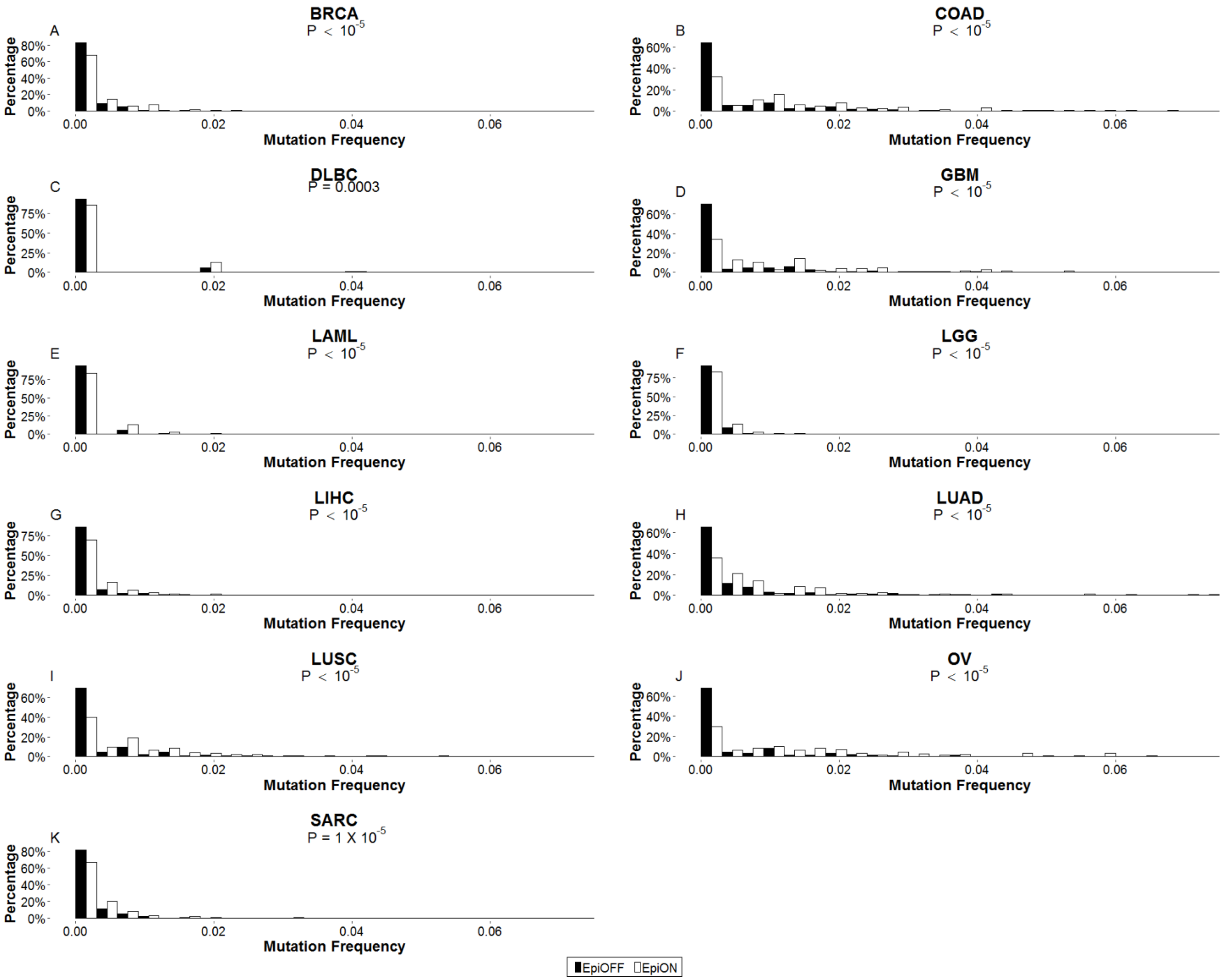

Figure S10. Tissue-specific distributions of mutation frequency of EpiON and EpiOFF genes after 50-fold subsampling

Legends and captions follow Fig 1.

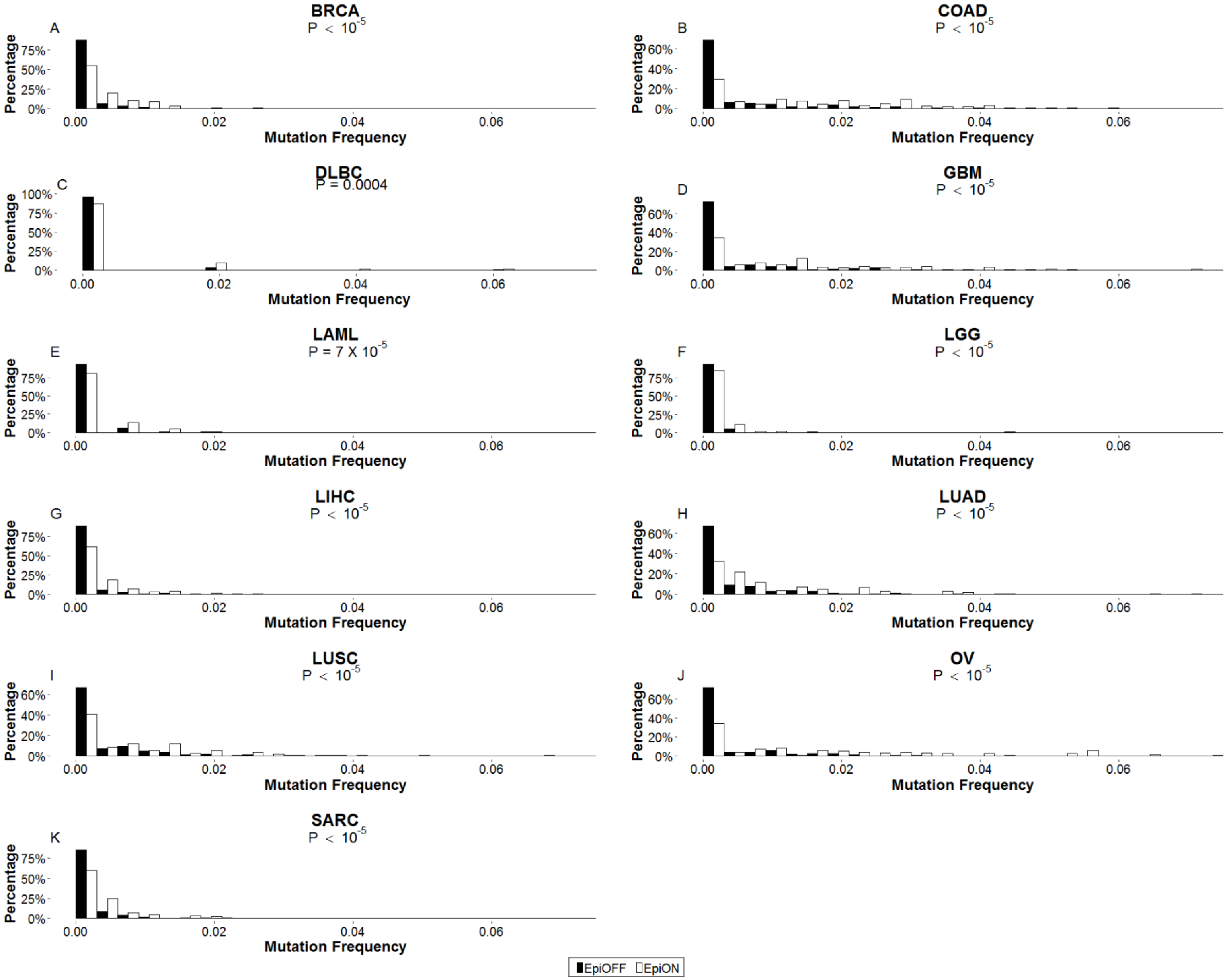

Figure S11. Tissue-specific distributions of mutation frequency of EpiON and EpiOFF genes after 100-fold subsampling

Legends and captions follow Fig 1.

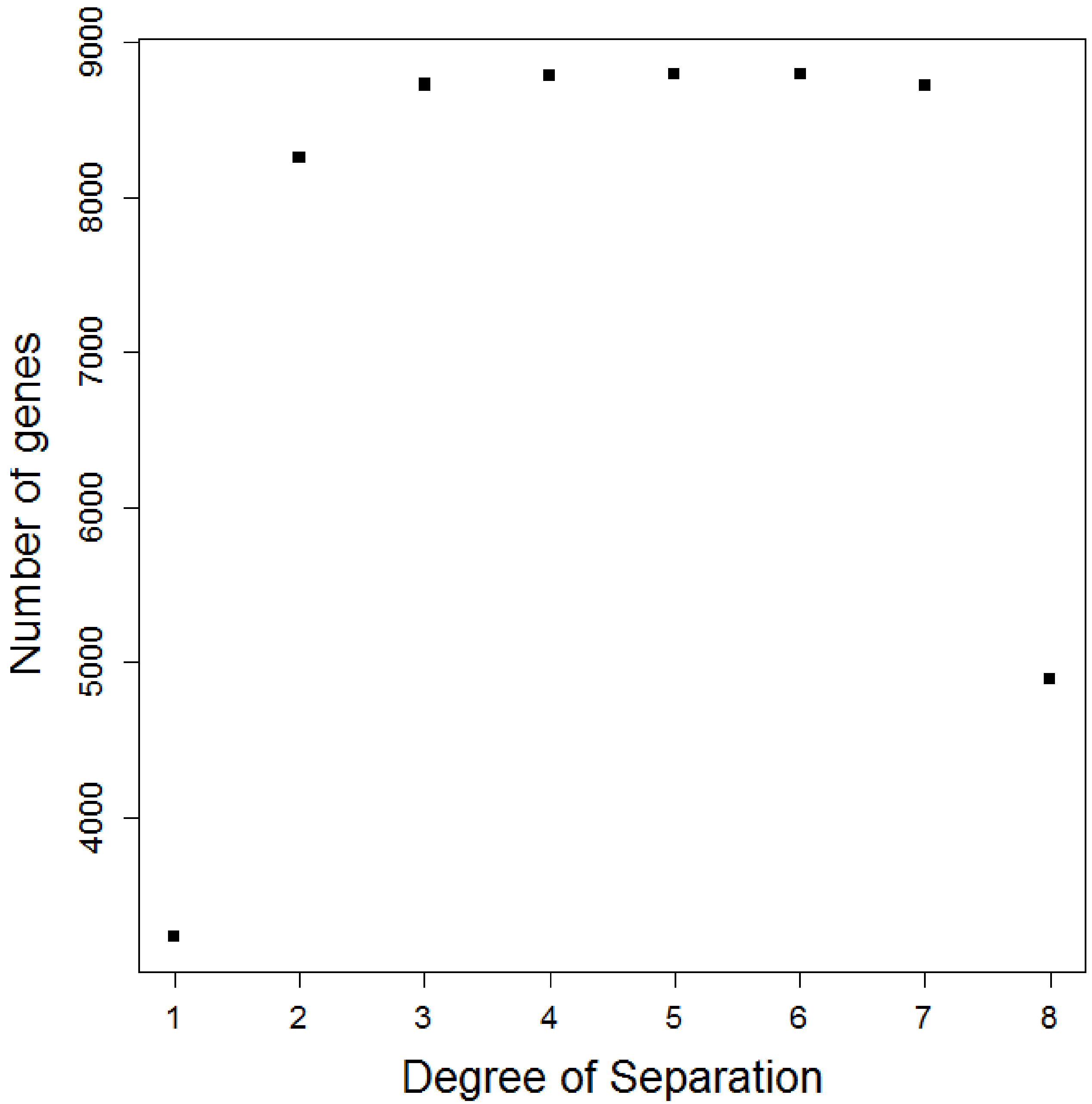

Figure S12. Number of genes with at least 5 PPI n^{th}-degree neighbors

The figure shows the number of genes with at least 5 PPI neighbors in degree of separation varying from 1 to 8.

Supplementary tables

Feature	Total Number of Genes	Total Number Significantly Associated Genes	Number of Significant Positively Associated Genes	Fraction of Significant Positively Associated Genes
Chromatin State	91	11	8	0.7272
Expression	432	98	86	0.8776
Pathway	119	40	37	0.9250
Protein-protein Interaction	254	50	47	0.9400
Control Feature	251	23.9700±4.7810	14.0200±3.7118	0.5848±0.1549

Table S1. Directionality of the association between biological features with mutation frequency

The table shows the fraction of significant genes ($p < 0.03$) positively associated with each feature. Positively associated genes are genes with positive association between the feature under study and mutation frequency.

**Feature**	**Total Number of Significant Genes**	**Directionality of Association**	**Total Number of Significantly Associated Genes**	**Genes**
Chromatin State	11	Positive	8	CD79A
				CDH1
				DMD
				DYNC1H1
				IRF4
				NBEA
				PKDREJ
				PTEN
		Negative	3	FAT3
				MAGI2
				SCN9A
Expression	98	Positive	86	ABCA13
				ADAMTS12
				ADGRG4
				ALB
				ALMS1
				ARNT
				ATRX
				B2M
				BTG2
				C3
				CALD1
				CARD11
				CCDC168
				CD79A
				CEP250
				CIITA
				COL16A1
				CSDE1
				CSMD3
				DCHS2
				DNAH10
				DNAH11
				DNAH2
				DNAH3

				DNAH7
				DNAH9
				DOCK7
				EIF1AX
				EPHB4
				ERICH3
				FAM27E3
				FLG2
				FOXA1
				FOXP4
				GATA3
				HELZ2
				HUWE1
				HYDIN
				IGHG1
				IGHG2
				IRF4
				KAT6B
				KLHL6
				KMT2A
				KMT2C
				LRP1
				LRRK2
				MDN1
				MEF2B
				MSNP1
				MT-CYB
				MT-ND4
				MUC16
				MUC2
				MUC5AC
				MUC5B
				NALCN
				NBEA
				NCOR1
				P2RY8
				PCDH15
				PCDH17

				PCDHB12
				PIK3CA
				PIK3R1
				PRDM1
				PRKDC
				PRPF8
				RBBP6
				RP1
				RUFY3
				RUNX1
				SNRNP200
				SOCS1
				SPOP
				SPTA1
				SRCAP
				SRRM2
				SUPT16H
				TET1
				TMSB4X
				TP53
				TRRAP
				TSHZ3
				WDR87
				XIST
		Negative	12	ATAD5
				ATG2B
				BIRC6
				CNOT1
				CTNNB1
				ESPL1
				INTS2
				RXRB
				SCN9A
				TNRC6A
				VCAN
				ZNF629
Pathway	50	Positive	47	APC
				ATM

				AXIN1
				BCAR1
				C3
				CACNA1C
				CACNA1D
				COL4A6
				COL6A3
				CREBBP
				CTNNB1
				DMD
				ERBB4
				ESPL1
				FLT3
				GRM3
				ITPKB
				ITPR1
				ITPR3
				KRAS
				LRP2
				MTOR
				NOTCH1
				PIK3CA
				PIK3R1
				PIKFYVE
				PRKDC
				PTEN
				RANBP2
				RB1
				RELN
				RUNX1
				RYR2
				SOCS1
				SPOP
				TP53
				VHL
		Negative	3	BRAF
				CHRM3
				KIT

Protein-protein Interaction	50	Positive	47	ABCA2
				AKAP13
				ALB
				APOB
				ARID1B
				ARNT
				ATXN10
				BRAF
				CACNA1C
				CACNA1D
				CACNA1E
				CARD11
				CD79A
				COL4A6
				COL6A3
				EIF1AX
				ERBB4
				FAT4
				FBXW7
				FOXA1
				FUBP1
				IRF4
				LRP1
				LRP1B
				MUC16
				NCOR1
				NEB
				NIPBL
				NRXN1
				PBRM1
				PRDM1
				PRKDC
				PTPN14
				RAI1
				RDX
				RUNX1
				RXRB
				RYR3

				SOX9
				SPHKAP
				SPTBN4
				STK11
				SUPT16H
				TRRAP
				UBR5
				USH2A
				ZFHX3
		Negative	3	CEP350
				ITPR1
				KIT

Table S2. List of significantly associated genes

The table shows significantly associated genes for each feature and their directionality of association.

Feature	Total Number of Genes	Significant Positively Associated Genes				Significant Negatively Associated Genes			
		Total Number of Genes	OG	TSG	CANGene	Total Number of Genes	OG	TSG	CANGene
Chromatin State	91	8	2	3	4	3	0	0	0
Expression	432	86	11	10	23	12	1	0	1
Pathway	119	37	11	12	21	3	2	0	2
Protein-Protein Interaction	254	47	9	10	21	3	1	0	1

Table S3. Directionality of association and cancer genes

The table shows the number of cancer genes identified as significantly associated with each feature, either in positive or negative direction.
OG = Oncogene, TSG = Tumor Suppressor Gene, CANGene = Cancer Gene
OG and TSG assignment were based on classification by COSMIC. Some genes are labeled as both OG and TSG. Some genes are not labeled as either OG or TSG.

Feature	Directionality of Association	Gene Set Name	# Genes in Gene Set (K)	# Genes in Overlap (k)	k/K	p-value	FDR q-value
Chromatin State	Positive	REACTOME_IMMUNE_SYSTEM	933	5	0.0054	1.82E-07	2.86E-03
		REACTOME_ADAPTIVE_IMMUNE_SYSTEM	539	4	0.0074	1.26E-06	9.95E-03
		GSE41176_UNSTIM_VS_ANTI_IGM_STIM_TAK1_KO_BCELL_6H_UP	200	3	0.015	4.47E-06	2.35E-02
		BIOCARTA_CTCF_PATHWAY	23	2	0.087	6.70E-06	2.64E-02
		GO_MEMBRANE_MICRODOMAIN	288	3	0.0104	1.33E-05	4.20E-02
		GRESHOCK_CANCER_COPY_NUMBER_UP	323	3	0.0093	1.88E-05	4.93E-02
Expression	Positive	GO_REGULATION_OF_IMMUNE_SYSTEM_PROCESS	1403	18	0.0128	1.12E-11	9.32E-08
		GO_POSITIVE_REGULATION_OF_IMMUNE_SYSTEM_PROCESS	867	15	0.0173	1.18E-11	9.32E-08
		GO_ADENYL_NUCLEOTIDE_BINDING	1514	18	0.0119	3.87E-11	2.03E-07
		GO_DYNEIN_COMPLEX	43	6	0.1395	9.71E-11	3.83E-07
		GO_POSITIVE_REGULATION_OF_GENE_EXPRESSION	1733	18	0.0104	3.41E-10	9.23E-07
		GO_CILIUM	469	11	0.0235	3.51E-10	9.23E-07
		GO_CELL_PROJECTION	1786	18	0.0101	5.50E-10	1.24E-06
		GO_RIBONUCLEOTIDE_BINDING	1860	18	0.0097	1.05E-09	1.89E-06
		GO_MICROTUBULE_BASED_PROCESS	522	11	0.0211	1.08E-09	1.89E-06
		GO_ANTIGEN_RECEPTOR_MEDIATED_SIGNALING_PATHWAY	195	8	0.041	1.34E-09	2.11E-06
		WEST_ADRENOCORTICAL_TUMOR_DN	546	11	0.0201	1.72E-09	2.46E-06
		GO_MOVEMENT_OF_CELL_OR_SUBCELLULAR_COMPONENT	1275	15	0.0118	2.39E-09	3.14E-06
		GO_MICROTUBULE_MOTOR_ACTIVITY	77	6	0.0779	3.61E-09	4.38E-06
		GO_REGULATION_OF_TRANSCRIPTION_FROM_RNA_POLYMERASE_II_PROMOTER	1784	17	0.0095	4.16E-09	4.68E-06

		GO_MICROTUBULE_ASSOCIATED_COMPLEX	145	7	0.0483	4.90E-09	5.15E-06
		DODD_NASOPHARYNGEAL_CARCINOMA_UP	1821	17	0.0093	5.64E-09	5.56E-06
		GO_CILIARY_PLASM	85	6	0.0706	6.59E-09	6.12E-06
		GO_REGULATION_OF_CELL_ACTIVATION	484	10	0.0207	7.85E-09	6.88E-06
		GO_HYDROLASE_ACTIVITY_ACTING_ON_ACID_ANHYDRIDES	820	12	0.0146	1.05E-08	8.71E-06
		GO_POSITIVE_REGULATION_OF_CYTOKINE_PRODUCTION	370	9	0.0243	1.14E-08	9.01E-06
		GO_CHROMATIN_ORGANIZATION	663	11	0.0166	1.27E-08	9.55E-06
		GO_BIOLOGICAL_ADHESION	1032	13	0.0126	1.42E-08	1.02E-05
		GO_TRANSCRIPTION_FACTOR_BINDING	524	10	0.0191	1.66E-08	1.13E-05
		GO_REGULATION_OF_IMMUNE_RESPONSE	858	12	0.014	1.73E-08	1.13E-05
		GO_MULTICELLULAR_ORGANISMAL_HOMEOSTASIS	272	8	0.0294	1.81E-08	1.14E-05
		GO_POSITIVE_REGULATION_OF_IMMUNE_RESPONSE	563	10	0.0178	3.25E-08	1.90E-05
		GO_REGULATION_OF_CYTOKINE_PRODUCTION	563	10	0.0178	3.25E-08	1.90E-05
		GO_HOMEOSTATIC_PROCESS	1337	14	0.0105	3.72E-08	2.05E-05
		GO_ACTIVATION_OF_IMMUNE_RESPONSE	427	9	0.0211	3.90E-08	2.05E-05
		GO_ATPASE_ACTIVITY	427	9	0.0211	3.90E-08	2.05E-05
		GO_IMMUNE_SYSTEM_DEVELOPMENT	582	10	0.0172	4.43E-08	2.22E-05
		GSE8835_CD4_VS_CD8_TCELL_UP	200	7	0.035	4.51E-08	2.22E-05
		GO_POSITIVE_REGULATION_OF_CELL_ACTIVATION	311	8	0.0257	5.09E-08	2.43E-05
		GO_MICROTUBULE_BASED_MOVEMENT	205	7	0.0341	5.34E-08	2.48E-05
		BIOCARTA_CTCF_PATHWAY	23	4	0.1739	5.97E-08	2.60E-05
		GO_AXONEME_PART	23	4	0.1739	5.97E-08	2.60E-05
		GO_LYMPHOCYTE_DIFFERENTIATION	209	7	0.0335	6.10E-08	2.60E-05
		GO_IMMUNE_RESPONSE_REGULATING_CELL_SURFACE_RECEPTOR_SIGNALING_PATHWAY	323	8	0.0248	6.80E-08	2.82E-05
		REACTOME_TERMINATION_OF_O_GLYCAN_BIOSYNTHESIS	24	4	0.1667	7.15E-08	2.89E-05

		PID_IL4_2PATHWAY	65	5	0.0769	8.27E-08	3.26E-05
		GO_REGULATION_OF_MULTICELLULAR_ORGANISMAL_DEVELOPMENT	1672	15	0.009	8.57E-08	3.29E-05
		GO_MOTOR_ACTIVITY	131	6	0.0458	8.87E-08	3.33E-05
		GO_RNA_POLYMERASE_II_TRANSCRIPTION_FACTOR_ACTIVITY_SEQUENCE_SPECIFIC_DNA_BINDING	629	10	0.0159	9.12E-08	3.34E-05
		GO_POSITIVE_REGULATION_OF_TRANSCRIPTION_FROM_RNA_POLYMERASE_II_PROMOTER	1004	12	0.012	9.52E-08	3.41E-05
		GO_CHROMOSOME_ORGANIZATION	1009	12	0.0119	1.00E-07	3.52E-05
		SENGUPTA_NASOPHARYNGEAL_CARCINOMA_DN	349	8	0.0229	1.23E-07	4.21E-05
		GO_CATALYTIC_COMPLEX	1038	12	0.0116	1.36E-07	4.57E-05
		GO_REGULATION_OF_CELL_DIFFERENTIATION	1492	14	0.0094	1.42E-07	4.68E-05
		BIOCARTA_VEGF_PATHWAY	29	4	0.1379	1.59E-07	5.11E-05
		GO_POSITIVE_REGULATION_OF_BIOSYNTHETIC_PROCESS	1805	15	0.0083	2.29E-07	7.15E-05
		GO_ADAPTIVE_IMMUNE_RESPONSE_BASED_ON_SOMATIC_RECOMBINATION_OF_IMMUNE_RECEPTORS_BUILT_FROM_IMMUNOGLOBULIN_SUPERFAMILY_DOMAINS	154	6	0.039	2.31E-07	7.15E-05
		TCGA_GLIOBLASTOMA_MUTATED	8	3	0.375	2.42E-07	7.34E-05
		GO_CHROMATIN_MODIFICATION	539	9	0.0167	2.79E-07	8.29E-05
		GO_MICROTUBULE	405	8	0.0198	3.80E-07	1.11E-04
		GO_TISSUE_HOMEOSTASIS	171	6	0.0351	4.28E-07	1.23E-04
		GO_POSITIVE_REGULATION_OF_MULTICELLULAR_ORGANISMAL_PROCESS	1395	13	0.0093	4.55E-07	1.28E-04
		GO_ANATOMICAL_STRUCTURE_HOMEOSTASIS	285	7	0.0246	4.97E-07	1.37E-04
		GO_ANATOMICAL_STRUCTURE_FORMATION_INVOLVED_IN_MORPHOGENESIS	957	11	0.0115	5.03E-07	1.37E-04
		GO_REGULATION_OF_ORGANELLE_ORGANIZATION	1178	12	0.0102	5.22E-07	1.38E-04
		GO_POSITIVE_REGULATION_OF_RESPONSE_TO_STIMULUS	1929	15	0.0078	5.31E-07	1.38E-04
		GO_ADAPTIVE_IMMUNE_RESPONSE	288	7	0.0243	5.33E-07	1.38E-04
		GO_LEUKOCYTE_DIFFERENTIATION	292	7	0.024	5.85E-07	1.49E-04

		GO_CYTOSKELETAL_PART	1436	13	0.0091	6.29E-07	1.57E-04
		GO_CYTOSKELETON	1967	15	0.0076	6.78E-07	1.67E-04
		GO_IMMUNE_SYSTEM_PROCESS	1984	15	0.0076	7.55E-07	1.79E-04
		GO_PROTEIN_O_LINKED_GLYCOSYLATION	101	5	0.0495	7.57E-07	1.79E-04
		GO_CELL_CELL_ADHESION	608	9	0.0148	7.59E-07	1.79E-04
		GO_ENZYME_BINDING	1737	14	0.0081	8.81E-07	2.04E-04
		GO_REGULATION_OF_HEMOPOIESIS	314	7	0.0223	9.50E-07	2.17E-04
		GO_THYMOCYTE_AGGREGATION	45	4	0.0889	9.77E-07	2.20E-04
		GO_REGULATORY_REGION_NUCLEIC_ACID_BINDING	818	10	0.0122	1.00E-06	2.22E-04
		LEE_DIFFERENTIATING_T_LYMPHOCYTE	200	6	0.03	1.07E-06	2.34E-04
		GRESHOCK_CANCER_COPY_NUMBER_UP	323	7	0.0217	1.15E-06	2.47E-04
		GO_CELL_MOTILITY	835	10	0.012	1.20E-06	2.56E-04
		GO_REGULATION_OF_INTERLEUKIN_2_PRODUCTION	48	4	0.0833	1.27E-06	2.67E-04
		GO_MICROTUBULE_CYTOSKELETON	1068	11	0.0103	1.46E-06	3.04E-04
		GO_RESPONSE_TO_EXTERNAL_STIMULUS	1821	14	0.0077	1.53E-06	3.14E-04
		GO_AXONEMAL_DYNEIN_COMPLEX	14	3	0.2143	1.56E-06	3.16E-04
		GO_CELLULAR_RESPONSE_TO_STRESS	1565	13	0.0083	1.64E-06	3.26E-04
		GO_LYMPHOCYTE_ACTIVATION	342	7	0.0205	1.67E-06	3.30E-04
		GO_INTRACELLULAR_SIGNAL_TRANSDUCTION	1572	13	0.0083	1.72E-06	3.34E-04
		GO_CILIUM_OR_FLAGELLUM_DEPENDENT_CELL_MOTILITY	15	3	0.2	1.95E-06	3.75E-04
		GO_NEGATIVE_REGULATION_OF_CELLULAR_COMPONENT_ORGANIZATION	684	9	0.0132	1.99E-06	3.77E-04
		GO_T_CELL_DIFFERENTIATION	123	5	0.0407	2.01E-06	3.77E-04
		GO_B_CELL_RECEPTOR_SIGNALING_PATHWAY	54	4	0.0741	2.05E-06	3.80E-04
		GO_LOCOMOTION	1114	11	0.0099	2.20E-06	4.03E-04
		GO_CELL_FATE_COMMITMENT	227	6	0.0264	2.23E-06	4.04E-04

		GO_CALCIUM_ION_BINDING	697	9	0.0129	2.32E-06	4.16E-04
		GO_POSITIVE_REGULATION_OF_BINDING	127	5	0.0394	2.35E-06	4.16E-04
		GO_CELL_PROJECTION_ORGANIZATION	902	10	0.0111	2.39E-06	4.19E-04
		GO_REGULATION_OF_LEUKOCYTE_DIFFERENTIATION	232	6	0.0259	2.53E-06	4.37E-04
		GO_RESPONSE_TO_OXYGEN_CONTAINING_COMPOUND	1381	12	0.0087	2.72E-06	4.64E-04
		GO_O_GLYCAN_PROCESSING	58	4	0.069	2.74E-06	4.64E-04
		BIOCARTA_ARF_PATHWAY	17	3	0.1765	2.91E-06	4.87E-04
		REACTOME_O_LINKED_GLYCOSYLATION_OF_MUCINS	59	4	0.0678	2.93E-06	4.87E-04
		GO_CELLULAR_RESPONSE_TO_DNA_DAMAGE_STIMULUS	720	9	0.0125	3.03E-06	4.97E-04
		GO_MACROMOLECULAR_COMPLEX_BINDING	1399	12	0.0086	3.10E-06	4.97E-04
		GO_POSITIVE_REGULATION_OF_CELL_ADHESION	376	7	0.0186	3.12E-06	4.97E-04
		GO_TRANSCRIPTION_FROM_RNA_POLYMERASE_II_PROMOTER	724	9	0.0124	3.16E-06	4.97E-04
		GO_NEUROGENESIS	1402	12	0.0086	3.17E-06	4.97E-04
	Negative	GO_REGULATION_OF_SISTER_CHROMATID_COHESION	17	2	0.1176	8.48E-06	3.42E-02
		GO_REGULATION_OF_CELL_CYCLE_PROCESS	558	4	0.0072	9.85E-06	3.42E-02
		GO_HORMONE_RECEPTOR_BINDING	168	3	0.0179	1.03E-05	3.42E-02
		GSE27241_WT_VS_RORGT_KO_TH17_POLARIZED_CD4_TCELL_TREATED_WITH_DIGOXIN_UP	170	3	0.0176	1.07E-05	3.42E-02
		GSE10240_CTRL_VS_IL17_AND_IL22_STIM_PRIMARY_BRONCHIAL_EPITHELIAL_CELLS_DN	200	3	0.015	1.74E-05	3.42E-02
		GSE360_L_DONOVANI_VS_T_GONDII_MAC_UP	200	3	0.015	1.74E-05	3.42E-02
		GSE360_T_GONDII_VS_M_TUBERCULOSIS_MAC_DN	200	3	0.015	1.74E-05	3.42E-02
		GSE7852_LN_VS_THYMUS_TCONV_DN	200	3	0.015	1.74E-05	3.42E-02
		KEGG_THYROID_CANCER	29	2	0.069	2.53E-05	4.10E-02
		GO_REGULATION_OF_GENE_EXPRESSION_EPIGENETIC	229	3	0.0131	2.60E-05	4.10E-02
		GO_REGULATION_OF_MYELINATION	33	2	0.0606	3.29E-05	4.71E-02

Pathway	Positive	KEGG_PATHWAYS_IN_CANCER	328	15	0.0457	3.74E-23	5.90E-19
		GO_REGULATION_OF_PHOSPHORUS_METABOLIC_PROCESS	1618	21	0.013	2.00E-21	1.57E-17
		GRESHOCK_CANCER_COPY_NUMBER_UP	323	13	0.0402	2.45E-19	1.29E-15
		GO_INTRACELLULAR_SIGNAL_TRANSDUCTION	1572	19	0.0121	1.24E-18	4.90E-15
		GO_REGULATION_OF_INTRACELLULAR_SIGNAL_TRANSDUCTION	1656	19	0.0115	3.25E-18	1.03E-14
		GO_REGULATION_OF_TRANSFERASE_ACTIVITY	946	16	0.0169	7.89E-18	2.07E-14
		GO_REGULATION_OF_CELLULAR_LOCALIZATION	1277	17	0.0133	2.98E-17	5.92E-14
		KEGG_PROSTATE_CANCER	89	9	0.1011	3.01E-17	5.92E-14
		KEGG_ENDOMETRIAL_CANCER	52	8	0.1538	5.75E-17	1.01E-13
		GO_REGULATION_OF_PROTEIN_MODIFICATION_PROCESS	1710	18	0.0105	1.54E-16	2.43E-13
		GO_REGULATION_OF_CELL_DEATH	1472	17	0.0115	3.12E-16	4.47E-13
		GO_REGULATION_OF_TRANSPORT	1804	18	0.01	3.91E-16	5.14E-13
		GO_HOMEOSTATIC_PROCESS	1337	16	0.012	1.75E-15	2.12E-12
		GO_POSITIVE_REGULATION_OF_INTRACELLULAR_SIGNAL_TRANSDUCTION	876	14	0.016	3.07E-15	3.46E-12
		GO_REGULATION_OF_IMMUNE_SYSTEM_PROCESS	1403	16	0.0114	3.68E-15	3.87E-12
		GO_ENZYME_BINDING	1737	17	0.0098	4.71E-15	4.64E-12
		GO_RESPONSE_TO_ENDOGENOUS_STIMULUS	1450	16	0.011	6.13E-15	5.69E-12
		TCGA_GLIOBLASTOMA_MUTATED	8	5	0.625	1.43E-14	1.19E-11
		GO_POSITIVE_REGULATION_OF_CELL_COMMUNICATION	1532	16	0.0104	1.43E-14	1.19E-11
		GO_REGULATION_OF_KINASE_ACTIVITY	776	13	0.0168	2.02E-14	1.59E-11
		GO_RESPONSE_TO_ABIOTIC_STIMULUS	1024	14	0.0137	2.58E-14	1.86E-11
		GO_POSITIVE_REGULATION_OF_RESPONSE_TO_STIMULUS	1929	17	0.0088	2.60E-14	1.86E-11
		DING_LUNG_CANCER_MUTATED_SIGNIFICANTLY	26	6	0.2308	4.05E-14	2.70E-11
		GO_IMMUNE_SYSTEM_PROCESS	1984	17	0.0086	4.10E-14	2.70E-11
		KEGG_COLORECTAL_CANCER	62	7	0.1129	5.71E-14	3.60E-11

	REACTOME_SIGNALING_BY_PDGF	122	8	0.0656	7.07E-14	4.29E-11
	GO_POSITIVE_REGULATION_OF_MULTICELLULAR_ORGANISMAL_PROCESS	1395	15	0.0108	7.95E-14	4.55E-11
	KEGG_GLIOMA	65	7	0.1077	8.08E-14	4.55E-11
	GO_POSITIVE_REGULATION_OF_GENE_EXPRESSION	1733	16	0.0092	9.50E-14	5.17E-11
	GO_CELL_PROLIFERATION	672	12	0.0179	1.15E-13	6.05E-11
	GO_RESPONSE_TO_HORMONE	893	13	0.0146	1.20E-13	6.09E-11
	GO_POSITIVE_REGULATION_OF_BIOSYNTHETIC_PROCESS	1805	16	0.0089	1.77E-13	8.72E-11
	GO_POSITIVE_REGULATION_OF_PROTEIN_METABOLIC_PROCESS	1492	15	0.0101	2.09E-13	9.71E-11
	GO_REGULATION_OF_CELL_DIFFERENTIATION	1492	15	0.0101	2.09E-13	9.71E-11
	KEGG_PHOSPHATIDYLINOSITOL_SIGNALING_SYSTEM	76	7	0.0921	2.52E-13	1.14E-10
	GO_REGULATION_OF_PROTEIN_LOCALIZATION	950	13	0.0137	2.62E-13	1.15E-10
	ST_ADRENERGIC	36	6	0.1667	3.40E-13	1.45E-10
	GO_CELL_DEATH	1001	13	0.013	5.05E-13	2.10E-10
	GO_KINASE_BINDING	606	11	0.0182	1.20E-12	4.86E-10
	REACTOME_DOWNSTREAM_SIGNAL_TRANSDUCTION	95	7	0.0737	1.26E-12	4.97E-10
	KEGG_TYPE_II_DIABETES_MELLITUS	47	6	0.1277	1.86E-12	7.17E-10
	REACTOME_SIGNALING_BY_ERBB2	101	7	0.0693	1.95E-12	7.34E-10
	GO_RESPONSE_TO_NITROGEN_COMPOUND	859	12	0.014	2.03E-12	7.46E-10
	GO_CELL_DEVELOPMENT	1426	14	0.0098	2.26E-12	7.94E-10
	GO_POSITIVE_REGULATION_OF_IMMUNE_SYSTEM_PROCESS	867	12	0.0138	2.27E-12	7.94E-10
	GO_NEGATIVE_REGULATION_OF_CELL_DEATH	872	12	0.0138	2.42E-12	8.31E-10
	GO_RESPONSE_TO_OXYGEN_LEVELS	311	9	0.0289	2.79E-12	9.35E-10
	GO_HEART_DEVELOPMENT	466	10	0.0215	2.85E-12	9.35E-10
	GO_POSITIVE_REGULATION_OF_CELLULAR_COMPONENT_ORGANIZATION	1152	13	0.0113	2.94E-12	9.47E-10
	KEGG_FOCAL_ADHESION	201	8	0.0398	4.03E-12	1.27E-09

	GO_RESPONSE_TO_ORGANIC_CYCLIC_COMPOUND	917	12	0.0131	4.35E-12	1.34E-09
	GO_HEAD_DEVELOPMENT	709	11	0.0155	6.48E-12	1.97E-09
	BIOCARTA_CTCF_PATHWAY	23	5	0.2174	8.50E-12	2.52E-09
	KEGG_ACUTE_MYELOID_LEUKEMIA	60	6	0.1	8.63E-12	2.52E-09
	GO_MOVEMENT_OF_CELL_OR_SUBCELLULAR_COMPONENT	1275	13	0.0102	1.04E-11	2.99E-09
	GO_CELLULAR_RESPONSE_TO_ENDOGENOUS_STIMULUS	1008	12	0.0119	1.30E-11	3.66E-09
	GO_REGULATION_OF_SISTER_CHROMATID_SEGREGATION	67	6	0.0896	1.71E-11	4.74E-09
	GO_POSITIVE_REGULATION_OF_PHOSPHORUS_METABOLIC_PROCESS	1036	12	0.0116	1.78E-11	4.85E-09
	GO_CIRCULATORY_SYSTEM_DEVELOPMENT	788	11	0.014	2.00E-11	5.35E-09
	BIOCARTA_GSK3_PATHWAY	27	5	0.1852	2.03E-11	5.35E-09
	PID_CDC42_PATHWAY	70	6	0.0857	2.25E-11	5.80E-09
	KEGG_MELANOMA	71	6	0.0845	2.45E-11	6.23E-09
	GO_IMMUNE_SYSTEM_DEVELOPMENT	582	10	0.0172	2.52E-11	6.30E-09
	GO_RESPONSE_TO_OXYGEN_CONTAINING_COMPOUND	1381	13	0.0094	2.81E-11	6.91E-09
	KEGG_CHRONIC_MYELOID_LEUKEMIA	73	6	0.0822	2.91E-11	7.06E-09
	REACTOME_SIGNALING_BY_SCF_KIT	78	6	0.0769	4.38E-11	1.05E-08
	GO_POSITIVE_REGULATION_OF_PROTEIN_MODIFICATION_PROCESS	1135	12	0.0106	5.10E-11	1.19E-08
	PID_MET_PATHWAY	80	6	0.075	5.12E-11	1.19E-08
	GO_MUSCLE_STRUCTURE_DEVELOPMENT	432	9	0.0208	5.20E-11	1.19E-08
	GO_NEGATIVE_REGULATION_OF_CELL_CYCLE	433	9	0.0208	5.31E-11	1.20E-08
	GO_POSITIVE_REGULATION_OF_DEVELOPMENTAL_PROCESS	1142	12	0.0105	5.47E-11	1.21E-08
	GO_REGULATION_OF_GROWTH	633	10	0.0158	5.71E-11	1.25E-08
	GO_CENTRAL_NERVOUS_SYSTEM_DEVELOPMENT	872	11	0.0126	5.88E-11	1.27E-08
	KEGG_SMALL_CELL_LUNG_CANCER	84	6	0.0714	6.91E-11	1.47E-08
	GO_CELLULAR_RESPONSE_TO_ORGANIC_SUBSTANCE	1848	14	0.0076	7.04E-11	1.48E-08

	GO_REGULATION_OF_CHROMOSOME_SEGREGATION	85	6	0.0706	7.43E-11	1.54E-08
	GO_REGULATION_OF_CELL_PROLIFERATION	1496	13	0.0087	7.53E-11	1.54E-08
	GO_TISSUE_DEVELOPMENT	1518	13	0.0086	9.01E-11	1.82E-08
	REACTOME_SIGNALING_BY_ERBB4	90	6	0.0667	1.05E-10	2.08E-08
	GO_REGULATION_OF_MITOTIC_CELL_CYCLE	468	9	0.0192	1.05E-10	2.08E-08
	KEGG_CALCIUM_SIGNALING_PATHWAY	178	7	0.0393	1.08E-10	2.11E-08
	REACTOME_IMMUNE_SYSTEM	933	11	0.0118	1.20E-10	2.31E-08
	GO_PHOSPHORYLATION	1228	12	0.0098	1.26E-10	2.36E-08
	REACTOME_PI3K_EVENTS_IN_ERBB4_SIGNALING	38	5	0.1316	1.26E-10	2.36E-08
	GO_CELLULAR_RESPONSE_TO_STRESS	1565	13	0.0083	1.31E-10	2.43E-08
	GO_PROTEIN_PHOSPHORYLATION	944	11	0.0117	1.36E-10	2.50E-08
	PID_IFNG_PATHWAY	40	5	0.125	1.65E-10	2.98E-08
	GO_REGULATION_OF_CELL_CYCLE_PHASE_TRANSITION	321	8	0.0249	1.68E-10	3.01E-08
	REACTOME_DOWNSTREAM_SIGNALING_OF_ACTIVATED_FGFR	100	6	0.06	2.01E-10	3.55E-08
	GO_NEGATIVE_REGULATION_OF_MITOTIC_CELL_CYCLE	199	7	0.0352	2.37E-10	4.15E-08
	REACTOME_PI3K_EVENTS_IN_ERBB2_SIGNALING	44	5	0.1136	2.71E-10	4.69E-08
	GO_TRANSCRIPTION_FACTOR_BINDING	524	9	0.0172	2.85E-10	4.88E-08
	SIG_CHEMOTAXIS	45	5	0.1111	3.05E-10	5.17E-08
	REACTOME_SIGNALING_BY_EGFR_IN_CANCER	109	6	0.055	3.39E-10	5.67E-08
	PID_PS1_PATHWAY	46	5	0.1087	3.42E-10	5.67E-08
	BIOCARTA_PS1_PATHWAY	14	4	0.2857	3.54E-10	5.81E-08
	REACTOME_ADAPTIVE_IMMUNE_SYSTEM	539	9	0.0167	3.65E-10	5.90E-08
	GO_REGULATION_OF_CELLULAR_COMPONENT_BIOGENESIS	767	10	0.013	3.67E-10	5.90E-08
	GO_NEGATIVE_REGULATION_OF_PHOSPHORUS_METABOLIC_PROCESS	541	9	0.0166	3.77E-10	6.01E-08
	GO_FOREBRAIN_DEVELOPMENT	357	8	0.0224	3.89E-10	6.13E-08

	Negative	GSE25088_ROSIGLITAZONE_VS_IL4_AND_ROSIGLITAZONE_STIM_MACROPHAGE_DAY10_DN	200	3	0.015	8.12E-08	1.28E-03
		GO_VISUAL_BEHAVIOR	50	2	0.04	3.48E-06	2.35E-02
		KEGG_ACUTE_MYELOID_LEUKEMIA	60	2	0.0333	5.02E-06	2.35E-02
		GO_SOMATIC_STEM_CELL_POPULATION_MAINTENANCE	66	2	0.0303	6.09E-06	2.35E-02
		GO_ASSOCIATIVE_LEARNING	73	2	0.0274	7.46E-06	2.35E-02
		GO_HEMATOPOIETIC_PROGENITOR_CELL_DIFFERENTIATION	98	2	0.0204	1.35E-05	3.54E-02
		GO_T_CELL_DIFFERENTIATION	123	2	0.0163	2.13E-05	4.00E-02
		GO_LEARNING	131	2	0.0153	2.41E-05	4.00E-02
		GO_MAINTENANCE_OF_CELL_NUMBER	132	2	0.0152	2.45E-05	4.00E-02
		GO_REGULATION_OF_SYNAPTIC_PLASTICITY	140	2	0.0143	2.76E-05	4.00E-02
		GO_POSITIVE_REGULATION_OF_MULTICELLULAR_ORGANISMAL_PROCESS	1395	3	0.0022	2.79E-05	4.00E-02
Protein-protein Interaction	Positive	GO_POSITIVE_REGULATION_OF_GENE_EXPRESSION	1733	20	0.0115	1.12E-16	1.77E-12
		GO_CIRCULATORY_SYSTEM_DEVELOPMENT	788	14	0.0178	3.43E-14	2.71E-10
		GO_REGULATION_OF_MULTICELLULAR_ORGANISMAL_DEVELOPMENT	1672	17	0.0102	3.08E-13	1.62E-09
		GO_REGULATION_OF_CELL_DIFFERENTIATION	1492	16	0.0107	8.19E-13	3.23E-09
		GO_POSITIVE_REGULATION_OF_MULTICELLULAR_ORGANISMAL_PROCESS	1395	15	0.0108	4.80E-12	1.32E-08
		GO_POSITIVE_REGULATION_OF_DEVELOPMENTAL_PROCESS	1142	14	0.0123	5.04E-12	1.32E-08
		GO_ENZYME_BINDING	1737	16	0.0092	8.03E-12	1.81E-08
		GO_NEUROGENESIS	1402	14	0.01	7.56E-11	1.49E-07
		GO_POSITIVE_REGULATION_OF_BIOSYNTHETIC_PROCESS	1805	15	0.0083	1.77E-10	3.10E-07
		GO_NEGATIVE_REGULATION_OF_DEVELOPMENTAL_PROCESS	801	11	0.0137	4.15E-10	6.54E-07
		GO_REGULATORY_REGION_NUCLEIC_ACID_BINDING	818	11	0.0134	5.17E-10	7.41E-07
		GO_NEURON_DIFFERENTIATION	874	11	0.0126	1.03E-09	1.36E-06
		GO_REGULATION_OF_TRANSCRIPTION_FROM_RNA_POLYMERASE_II_PROMOTER	1784	14	0.0078	1.72E-09	2.09E-06
		GO_REGULATION_OF_TRANSPORT	1804	14	0.0078	1.98E-09	2.23E-06

		GO_REGULATION_OF_PROTEIN_LOCALIZATION	950	11	0.0116	2.46E-09	2.58E-06
		GO_TRANSCRIPTION_FROM_RNA_POLYMERASE_II_PROMOTER	724	10	0.0138	2.71E-09	2.67E-06
		GO_REGULATION_OF_CELLULAR_LOCALIZATION	1277	12	0.0094	4.29E-09	3.97E-06
		GO_CHROMOSOME_ORGANIZATION	1009	11	0.0109	4.58E-09	4.01E-06
		GO_BIOLOGICAL_ADHESION	1032	11	0.0107	5.78E-09	4.80E-06
		GO_CELL_DEVELOPMENT	1426	12	0.0084	1.46E-08	1.15E-05
		GO_CENTRAL_NERVOUS_SYSTEM_DEVELOPMENT	872	10	0.0115	1.58E-08	1.18E-05
		GO_HEART_DEVELOPMENT	466	8	0.0172	2.34E-08	1.68E-05
		GO_REGULATION_OF_CELL_PROLIFERATION	1496	12	0.008	2.47E-08	1.69E-05
		GO_REGULATION_OF_ACTIN_FILAMENT_BASED_PROCESS	312	7	0.0224	3.10E-08	2.03E-05
		GO_HEAD_DEVELOPMENT	709	9	0.0127	3.80E-08	2.40E-05
		GO_POSITIVE_REGULATION_OF_RESPONSE_TO_STIMULUS	1929	13	0.0067	4.46E-08	2.70E-05
		GO_T_CELL_SELECTION	36	4	0.1111	5.52E-08	3.22E-05
		GO_TRANSCRIPTION_FACTOR_BINDING	524	8	0.0153	5.77E-08	3.25E-05
		GO_REGULATION_OF_NERVOUS_SYSTEM_DEVELOPMENT	750	9	0.012	6.13E-08	3.33E-05
		GO_LYMPHOCYTE_DIFFERENTIATION	209	6	0.0287	7.57E-08	3.98E-05
		GO_HOMEOSTATIC_PROCESS	1337	11	0.0082	8.08E-08	4.11E-05
		GO_PROTEIN_KINASE_A_BINDING	42	4	0.0952	1.04E-07	5.05E-05
		GO_REGULATION_OF_PROTEIN_MODIFICATION_PROCESS	1710	12	0.007	1.06E-07	5.05E-05
		GO_CELL_FATE_COMMITMENT	227	6	0.0264	1.23E-07	5.71E-05
		GO_IMMUNE_SYSTEM_DEVELOPMENT	582	8	0.0137	1.28E-07	5.79E-05
		GO_POSITIVE_REGULATION_OF_CELL_DIFFERENTIATION	823	9	0.0109	1.34E-07	5.89E-05
		BENPORATH_ES_WITH_H3K27ME3	1118	10	0.0089	1.60E-07	6.80E-05
		GO_HIGH_VOLTAGE_GATED_CALCIUM_CHANNEL_ACTIVITY	11	3	0.2727	1.64E-07	6.82E-05
		GO_T_CELL_DIFFERENTIATION	123	5	0.0407	1.77E-07	6.96E-05

		GO_CELL_CELL_ADHESION	608	8	0.0132	1.79E-07	6.96E-05
		GO_RESPONSE_TO_ENDOGENOUS_STIMULUS	1450	11	0.0076	1.82E-07	6.96E-05
		DACOSTA_UV_RESPONSE_VIA_ERCC3_DN	855	9	0.0105	1.85E-07	6.96E-05
		GO_REGULATION_OF_INTRACELLULAR_TRANSPORT	621	8	0.0129	2.10E-07	7.71E-05
		GO_RNA_POLYMERASE_II_TRANSCRIPTION_FACTOR_ACTIVITY_SEQUENCE_SPECIFIC_DNA_BINDING	629	8	0.0127	2.32E-07	8.30E-05
		GO_POSITIVE_REGULATION_OF_PROTEIN_METABOLIC_PROCESS	1492	11	0.0074	2.42E-07	8.48E-05
		GO_REGULATION_OF_ORGANELLE_ORGANIZATION	1178	10	0.0085	2.58E-07	8.84E-05
		GO_EMBRYO_DEVELOPMENT	894	9	0.0101	2.69E-07	9.04E-05
		GO_TISSUE_DEVELOPMENT	1518	11	0.0072	2.87E-07	9.44E-05
		GO_NUCLEIC_ACID_BINDING_TRANSCRIPTION_FACTOR_ACTIVITY	1199	10	0.0083	3.03E-07	9.76E-05
		GO_POSITIVE_REGULATION_OF_CELL_COMMUNICATION	1532	11	0.0072	3.15E-07	9.92E-05
		GO_CHROMATIN_ORGANIZATION	663	8	0.0121	3.45E-07	1.07E-04
		GO_CELL_PROLIFERATION	672	8	0.0119	3.82E-07	1.16E-04
		GO_SINGLE_ORGANISM_CELL_ADHESION	459	7	0.0153	4.22E-07	1.26E-04
		GO_VASCULATURE_DEVELOPMENT	469	7	0.0149	4.87E-07	1.42E-04
		GO_CALCIUM_ION_BINDING	697	8	0.0115	5.02E-07	1.44E-04
		GO_LEUKOCYTE_DIFFERENTIATION	292	6	0.0205	5.39E-07	1.52E-04
		GO_REGULATION_OF_CYTOPLASMIC_TRANSPORT	481	7	0.0146	5.77E-07	1.60E-04
		GO_NEGATIVE_REGULATION_OF_MULTICELLULAR_ORGANISMAL_PROCESS	983	9	0.0092	5.94E-07	1.61E-04
		PUJANA_BRCA1_PCC_NETWORK	1652	11	0.0067	6.62E-07	1.77E-04
		GO_REGULATION_OF_INTRACELLULAR_SIGNAL_TRANSDUCTION	1656	11	0.0066	6.78E-07	1.78E-04
		GO_POSITIVE_REGULATION_OF_TRANSCRIPTION_FROM_RNA_POLYMERASE_II_PROMOTER	1004	9	0.009	7.08E-07	1.83E-04
		GO_REGULATION_OF_CYTOSKELETON_ORGANIZATION	502	7	0.0139	7.67E-07	1.95E-04
		GO_NEGATIVE_REGULATION_OF_RESPONSE_TO_STIMULUS	1360	10	0.0074	9.52E-07	2.38E-04

		GRESHOCK_CANCER_COPY_NUMBER_UP	323	6	0.0186	9.69E-07	2.39E-04
		KEGG_CALCIUM_SIGNALING_PATHWAY	178	5	0.0281	1.11E-06	2.69E-04
		ONKEN_UVEAL_MELANOMA_UP	783	8	0.0102	1.20E-06	2.86E-04
		GO_CHROMATIN_MODIFICATION	539	7	0.013	1.23E-06	2.90E-04
		GO_POSITIVE_T_CELL_SELECTION	21	3	0.1429	1.32E-06	3.05E-04
		GO_LYMPHOCYTE_ACTIVATION	342	6	0.0175	1.35E-06	3.08E-04
		GRAESSMANN_APOPTOSIS_BY_DOXORUBICIN_DN	1781	11	0.0062	1.38E-06	3.11E-04
		GO_REGULATION_OF_CELLULAR_PROTEIN_LOCALIZATION	552	7	0.0127	1.44E-06	3.20E-04
		GO_POSITIVE_REGULATION_OF_CELLULAR_PROTEIN_LOCALIZATION	360	6	0.0167	1.82E-06	3.97E-04
		GSE41867_LCMV_ARMSTRONG_VS_CLONE13_DAY8_EFFECTOR_CD8_TCELL_DN	199	5	0.0251	1.92E-06	4.13E-04
		GO_REGULATION_OF_CELL_DEVELOPMENT	836	8	0.0096	1.95E-06	4.13E-04
		GSE14308_TH1_VS_TH17_DN	200	5	0.025	1.96E-06	4.13E-04
		GO_KINASE_ACTIVITY	842	8	0.0095	2.05E-06	4.26E-04
		GO_DEVELOPMENTAL_PROCESS_INVOLVED_IN_REPRODUCTION	602	7	0.0116	2.55E-06	5.18E-04
		DING_LUNG_CANCER_MUTATED_SIGNIFICANTLY	26	3	0.1154	2.56E-06	5.18E-04
		GO_NEGATIVE_REGULATION_OF_CELL_DIFFERENTIATION	609	7	0.0115	2.75E-06	5.50E-04
		GO_GLAND_DEVELOPMENT	395	6	0.0152	3.10E-06	6.10E-04
		GO_RESPONSE_TO_HORMONE	893	8	0.009	3.17E-06	6.17E-04
		GO_INTRACELLULAR_SIGNAL_TRANSDUCTION	1572	10	0.0064	3.48E-06	6.68E-04
		GO_NEGATIVE_REGULATION_OF_CELL_PROLIFERATION	643	7	0.0109	3.93E-06	7.47E-04
		GO_LEUKOCYTE_ACTIVATION	414	6	0.0145	4.05E-06	7.60E-04
		GO_POSITIVE_REGULATION_OF_INTERLEUKIN_2_PRODUCTION	31	3	0.0968	4.42E-06	8.19E-04
		GO_REGULATION_OF_PHOSPHORUS_METABOLIC_PROCESS	1618	10	0.0062	4.49E-06	8.19E-04
		YAGI_AML_WITH_INV_16_TRANSLOCATION	422	6	0.0142	4.52E-06	8.19E-04
		GO_NEURON_PART	1265	9	0.0071	4.69E-06	8.40E-04

		GO_EPITHELIUM_DEVELOPMENT	945	8	0.0085	4.80E-06	8.50E-04
		GO_MUSCLE_STRUCTURE_DEVELOPMENT	432	6	0.0139	5.17E-06	9.05E-04
		GO_REPRODUCTION	1297	9	0.0069	5.73E-06	9.93E-04
		GO_NEGATIVE_REGULATION_OF_CELLULAR_COMPONENT_ORGANIZATION	684	7	0.0102	5.89E-06	1.01E-03
		GO_NEURON_DEVELOPMENT	687	7	0.0102	6.06E-06	1.03E-03
		GO_LEUKOCYTE_CELL_CELL_ADHESION	255	5	0.0196	6.41E-06	1.08E-03
		BLALOCK_ALZHEIMERS_DISEASE_UP	1691	10	0.0059	6.61E-06	1.10E-03
		GO_TRANSFERASE_ACTIVITY_TRANSFERRING_PHOSPHORUS_CONTAINING_GROUPS	992	8	0.0081	6.84E-06	1.12E-03
		GO_SKELETAL_SYSTEM_DEVELOPMENT	455	6	0.0132	6.94E-06	1.13E-03
		GO_CELLULAR_RESPONSE_TO_DNA_DAMAGE_STIMULUS	720	7	0.0097	8.21E-06	1.32E-03
		GO_REGULATION_OF_ANATOMICAL_STRUCTURE_MORPHOGENESIS	1021	8	0.0078	8.43E-06	1.34E-03
		GO_EXTRACELLULAR_MATRIX_COMPONENT	125	4	0.032	8.50E-06	1.34E-03
	Negative	HAMAI_APOPTOSIS_VIA_TRAIL_UP	584	3	0.0051	2.04E-06	3.22E-02

Table S4. Significantly enriched biological functions for each feature and directionality of association

The table shows biological functions significantly enriched for each feature and each directionality of association.

Genes	EpiAllON	EpiAllOFF	EpiDIFF
Genome	8030	27212	5739
Cancer genes	257*	130	228*
Oncogenes	60*	58	35*
Tumor Suppressor Genes	58*	39	34*

Table S5A. Number of Cancer Genes and its distribution in the genome based on chromatin state within tissues

Genes	EpiAllON	EpiAllOFF	EpiDIFF
Top Mutated	180	161	91
Cancer Genes	41	25	24
Oncogenes	12	7	9
Tumor Suppressor Genes	17	9	16*

Table S5B. Number of Cancer Genes in top mutated genes group based on chromatin state across tissues

The tables show the number of genes in different categories: Genome (all genes) [S5A]/Top Mutated genes [S5B], cancer genes, and its subcategories oncogenes and tumor suppressor genes, further subdivided into 3 different groups based on their chromatin state: EpiON (chromatin accessible across all normal tissues), EpiOFF (chromatin inaccessible across all normal tissues) and EpiDIFF (chromatin accessible in some normal tissues). Numbers marked by asterisks denote significant enrichment according to the hypergeometric test.

**Cancer Type**	**Epigenome Identifier (EID)**	**Sample Type**
BRCA	E027	Breast Myoepithelial Primary Cells
	E028	Breast variant Human Mammary Epithelial Cells (vHMEC)
	E119	HMEC Mammary Epithelial Primary Cells
COAD	E075	Colonic Mucosa
	E076	Colon Smooth Muscle
	E106	Sigmoid Colon
DLBC	E031	Primary B cells from cord blood
	E032	Primary B cells from peripheral blood
GBM/LGG	E053	Cortex derived primary cultured neurospheres
	E054	Ganglion Eminence derived primary cultured neurospheres
	E067	Brain Angular Gyrus
	E068	Brain Anterior Caudate
	E069	Brain Cingulate Gyrus
	E070	Brain Germinal Matrix
	E071	Brain Hippocampus Middle
	E072	Brain Inferior Temporal Lobe
	E073	Brain Dorsolateral Prefrontal Cortex
	E074	Brain Substantia Nigra
LAML	E029	Primary monocytes from peripheral blood
	E030	Primary neutrophils from peripheral blood
LIHC	E066	Liver
LUAD/LUSC	E096	Lung
	E128	NHLF Lung Fibroblast Primary Cells
OV	E097	Ovary
SARC	E100	Psoas Muscle
	E107	Skeletal Muscle Male
	E108	Skeletal Muscle Female
	E126	NHDF-Ad Adult Dermal Fibroblast Primary Cells
	E129	Osteoblast Primary Cells

Table S6A. Mapping of normal tissues from the Roadmap data to corresponding cancer types

Normal Tissue	Corresponding Cancer Type
Adipose	SARC
Adrenal	ACC/PCPG
Brain	GBM/LGG
Breast	BRCA
Colon	COAD
Kidney	KICH/KIRC/KIRP
Leukocyte	LAML
Liver	LIHC
Lung	LUAD/LUSC
Lymph node	DLBC
Ovary	OV
Prostate	PRAD
Skeletal muscle	SARC
Testis	TGCT
Thyroid	THCA

Table S6B. Mapping of normal tissues from Illumina Body Map 2.0 to corresponding cancer types

The table shows the mapping of normal tissues from Illumina Body Map 2.0 and the Roadmap data to corresponding TCGA cancer types. Note that some tissues, such as kidney and lung are mapped to multiple cancer types, and SARC is mapped to two normal tissues.

**Cancer**	**PPermutTwoSided**	**PPermutAlternative**
BRCA	<1e-5	0.99363
COAD	<1e-5	0.98928
DLBC	<1e-5	0.97314
LGG	<1e-5	0.99028
LAML	<1e-5	1
GBM	<1e-5	0.91017
LIHC	<1e-5	0.98165
LUAD	<1e-5	0.98804
LUSC	<1e-5	0.98724
OV	<1e-5	0.99194
SARC	<1e-5	0.96063

Table S7. Alternative permutation P-values for distribution of EpiON and EpiOFF genes

The table shows the permutation p-values for distributions of EpiON and EpiOFF genes using two-sided KS test (PPermutTwoSided), and one-sided KS test with the opposite direction compared to Figure 1 (PPermutAlternative).
